# Advantages of PROTACs in achieving selective degradation of homologous protein families

**DOI:** 10.3762/bjoc.22.49

**Published:** 2026-04-27

**Authors:** Luxi Yang, Xinfei Mao, Jingyi Zhang, Jing Shu, Wenhai Huang, Xiaowu Dong, Yinqiao Chen, Mingfei Wu

**Affiliations:** 1 Affiliated Yongkang First People’s Hospital, School of Pharmaceutical Sciences, Hangzhou Medical College, Zhejiang, Hangzhou, 311399, China; 2 Center of Safety Evaluation and Research, Hangzhou Medical College, Hangzhou 310053, China; 3 College of Pharmaceutical Sciences, Zhejiang University, Hangzhou, 310058, China

**Keywords:** homologous protein, PROTAC, protein–protein interaction, selectivity, ubiquitination

## Abstract

Proteolysis-targeting chimeras (PROTACs) have emerged as a promising therapeutic modality and now represent an important addition to the toolkit of medicinal chemists. Compared with conventional small-molecule inhibitors, PROTACs exhibit notable advantages, particularly in achieving selectivity within highly homologous proteins. The ability to discriminate among members of such families holds broad implications for future disease treatment. In this review, we primarily summarize the advantages of PROTACs in conferring selectivity toward highly homologous proteins. This focus will provide a feasible strategy for developing PROTACs that selectively target highly homologous proteins and will ultimately support future therapeutic applications.

## Introduction

The cell is the fundamental unit of structure and function in the human body [1–2]. More than 20,000 proteins act in concert to regulate the entire cellular life process [1]. To date, dysregulated protein function has been recognized as one of the leading causes of human diseases [3]. Although small-molecule inhibitors [4] remain the most widely used therapeutic agents for the treatment of disorders associated with abnormal protein activity, nearly all disease-relevant scaffolding proteins [5], transcription factors [6], and other non-enzymatic proteins [7] are essentially undruggable using conventional inhibitor-based approaches [4,8]. Consequently, protein–protein interaction (PPI)-based targeted protein degradation (TPD) strategies have attracted increasing attention over the past two decades [9–10]. Among them, PROTACs, one of the most extensively studied and promising TPD approaches, are reshaping the paradigm of small-molecule drug development [11].

**Figure 1:**
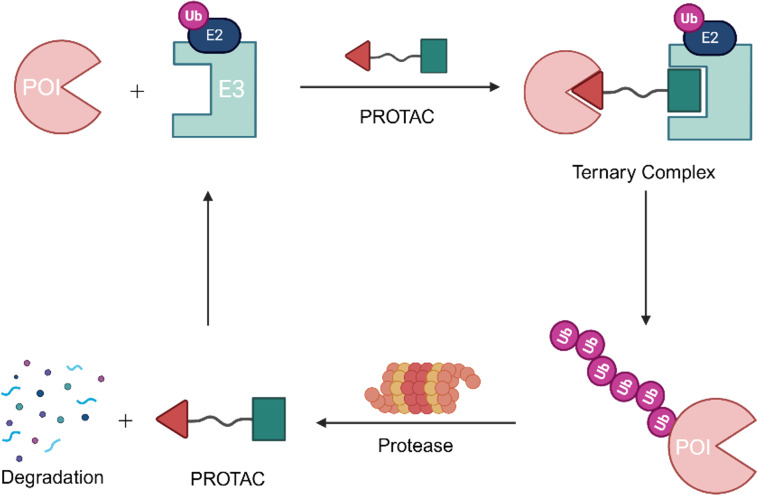
Mechanism of a PROTAC-mediated targeted protein degradation. Created in BioRender. Wu, M. (2026) https://BioRender.com/xig7tb2. This content is not subject to CC BY 4.0.

The PROTAC technology was initially conceptualized by Crews et al. in 2001 [11]. As a heterobifunctional molecule, a PROTAC comprises two distinct ligands: one targeting an E3 ubiquitin ligase and the other binding to a protein of interest (POI), covalently joined by a flexible linker [12]. Upon cellular entry, the PROTAC molecule facilitates the formation of a ternary complex by simultaneously recruiting the POI and the E3 ligase (Figure 1). This proximity enables the proteasome to recognize the complex, subsequently degrading the target protein via the endogenous ubiquitin–proteasome system [13–14]. Notably, this mechanism does not necessitate prolonged occupancy of the POI binding site. Instead, the transient formation of the ternary complex is sufficient to trigger rapid ubiquitination [15–16]. Consequently, a PROTAC operates through an event-driven pharmacological paradigm, distinguishing itself from the traditional occupancy-driven model of small-molecule inhibitors [17]. Furthermore, PROTACs function catalytically, requiring only substoichiometric doses to achieve effective protein degradation [18].

Recent reviews have extensively detailed the principles, advancements, and therapeutic prospects of TPD, with a particular focus on PROTACs [15,19]. As understanding of this field matures, medicinal chemists are increasingly evaluating the comparative advantages and limitations of PROTACs relative to traditional small-molecule inhibitors [20]. A defining strength of the PROTAC technology lies in its superior selectivity for target proteins, especially within highly homologous proteins [21–23]. Selectivity remains a cornerstone of pharmacology; inhibitors that lack specificity may interfere with off-target biological functions, resulting in diminished therapeutic efficacy and unacceptable toxicity [24]. Consequently, maximizing compound selectivity is a primary objective in drug discovery [25–26]. In the context of PROTAC design, it is imperative to minimize the degradation of non-targeted proteins.

While conventional inhibitors often exhibit lacking selectivity towards proteins with high sequence similarity, PROTACs leverage their heterobifunctional structure to achieve exquisite selectivity [24,27]. This is accomplished by fine-tuning the conformation of the ternary complex through the strategic modification of the POI ligand, the linker, and the E3 ligase ligand. Several studies have successfully utilized this strategy to enhance disease treatment outcomes via the PROTAC technology [22,28–29].

The rapid progression of the PROTAC technology from an academic concept to clinical application underscores its transformative potential. In recent years, numerous PROTAC candidates have entered clinical trials, marking a significant milestone for the field [23]. Notably, multiple degraders targeting the androgen receptor (AR), such as ARV-110**,** ARV-766, and CC-94676, are currently being evaluated in patients with prostate cancer, demonstrating that the event-driven pharmacology of PROTACs translates into durable target knockdown in humans [30–32]. Beyond the AR, the clinical landscape is expanding to include targets discussed extensively in this review. For instance, selective degraders of CDK isoforms are advancing through early-phase trials [33], with molecules like BSJ-03-123 and others showing promise in hematologic malignancies by exploiting the subtle structural differences among the highly homologous CDK family members to achieve precision targeting [34]. Similarly, efforts to clinically translate PROTACs against epigenetic targets, such as BRD4, are gaining momentum, aiming to overcome the isoform selectivity challenges that have historically hindered small-molecule inhibitors in the BET family [35]. This wave of clinical validation not only confirms the unique advantages of PROTACs in addressing previously undruggable targets but also highlights the critical importance of understanding how linker composition, E3 ligase choice, and protein–protein interactions govern the exquisite selectivity required for clinical success [24,36].

Accordingly, this review emphasizes the advantages of PROTACs in achieving high selectivity among homologous proteins. We systematically summarize the correlations between PROTAC structural components, specifically the POI ligand, the linker, and the E3 ligand, and their role in dictating isoform-specific selectivity. Furthermore, we examine the influence of PPIs, a factor increasingly recognized for its critical contribution to the refined selectivity of PROTACs. The review also explores potential selectivity variations arising from the spatial distribution of ubiquitination-accessible lysine residues within homologous proteins. In conclusion, by synthesizing current knowledge and offering future perspectives on PROTAC selectivity, this work aims to provide a robust framework and feasible strategies for the rational design of degraders specifically targeting homologous proteins.

## Review

### Influence of linkers on the selectivity of PROTACs in highly homologous protein families

As previously established [37], a PROTAC molecule comprises three essential components: a POI ligand, a linker, and an E3 ligand. In drug design, POI ligands are typically derived from FDA-approved small-molecule inhibitors, candidates in clinical trials, or their structural analogs. Similarly, E3 ligands are engineered to target specific E3 ubiquitin ligases. For instance, pomalidomide and lenalidomide are widely employed to recruit the CRBN ligase [38]. Given the established nature of these ligands, initial optimization often prioritizes the linker fragment, as its length and composition are critical determinants of target selectivity [39]. Recent evidence underscores the pivotal role of the linker in promoting the assembly of "positive cooperative" ternary complexes [40], wherein the linker participates in specific molecular interactions within the complex [41–42]. These findings have profound implications for the rational design of PROTACs that exhibit isoform-specific selectivity among structurally related POIs [43]. Consequently, this section first examines the influence of linker architecture on the overall selectivity of PROTACs, utilizing several high-profile targets as illustrative examples.

#### CDK

Cyclin-dependent kinases (CDKs) are members of the serine/threonine (S/T) kinase subfamily comprising 21 CDK enzymes [44]. CDK1, 2, 3, 4, and 6 play a key role in cell cycle regulation. CDK7, 8, 9, and 11 are mainly involved in transcriptional regulation. However, the biological functions of CDK10, 11, 14–18, and 20 have not been fully elucidated [45–46]. CDKs are essential in cell proliferation, transcriptional activity, and neuronal activity [44]. In addition, disorders of these protein kinases often exist in cancer and nervous system diseases [47]. Palbociclib was the first small-molecule inhibitor approved by the FDA in 2015 to target CDK4/6 in the CDK family [48]. Soon after, ribociclib and abemaciclib, small-molecule inhibitors targeting CDK4/6, were also approved by the FDA for breast cancer treatment [49–50]. However, due to the high homology of the CDK family (for example, the homology of CDK4/6 is 71%, the homology of CDK2/3 is 76% [51], the homology of CDK8/19 is 97%) [52], small-molecule inhibitors may often inhibit several CDK members, thus, to a certain degree, cause off-target toxicity and reduce the therapeutic effect [53].

Since PROTACs have been proven to demonstrate significant advantages over small-molecule inhibitors in selectivity, more and more research has focused on how to design PROTACs that can target the degradation of the CDK family with high specificity in recent years [12]. The selectivity of PROTACs toward CDK4/6 over other highly homologous CDKs can be attributed to their event-driven pharmacology and the formation of stable ternary complexes that exploit subtle structural differences among the family members [54]. Unlike traditional kinase inhibitors that rely on sustained occupancy of conserved ATP-binding pockets, PROTACs induce transient yet specific protein–protein interactions that dictate ubiquitination efficiency. By fine-tuning the length and composition of the linker, as well as the choice of E3 ligase and its attachment geometry, PROTACs can achieve isoform-specific degradation. This approach not only minimizes off-target toxicity but also enables the targeting of CDK6 in contexts where CDK4 is functionally redundant, such as in Ph+ ALL.

**Figure 2:**
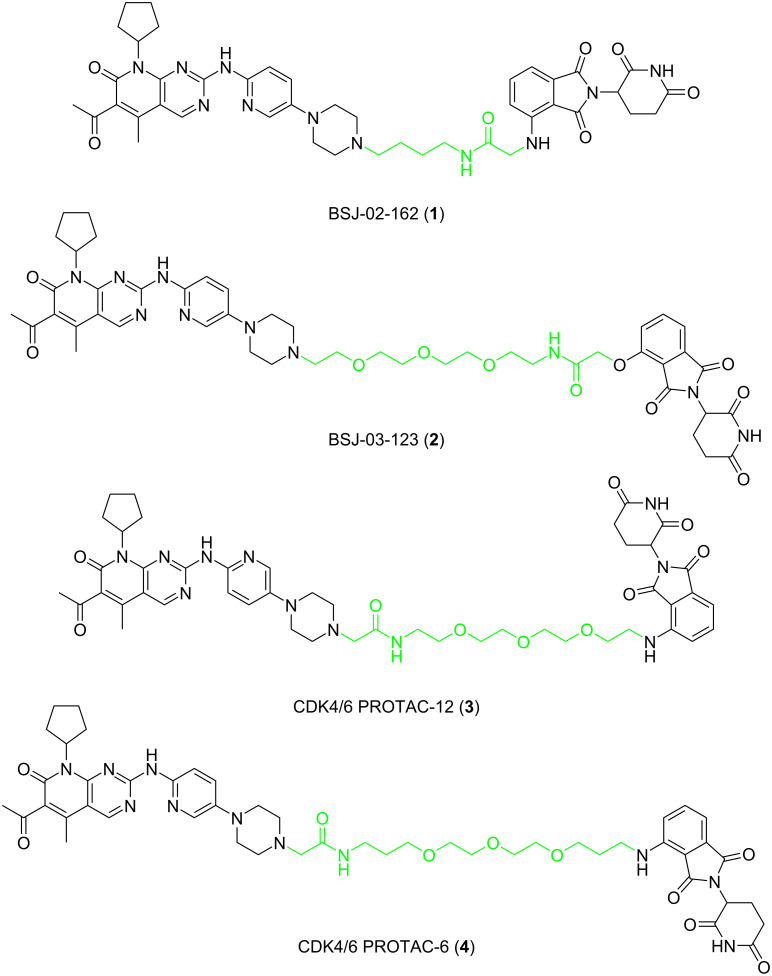
CDK4/6 PROTACs with alkyl or PEG chains as linkers.

**Figure 3:**
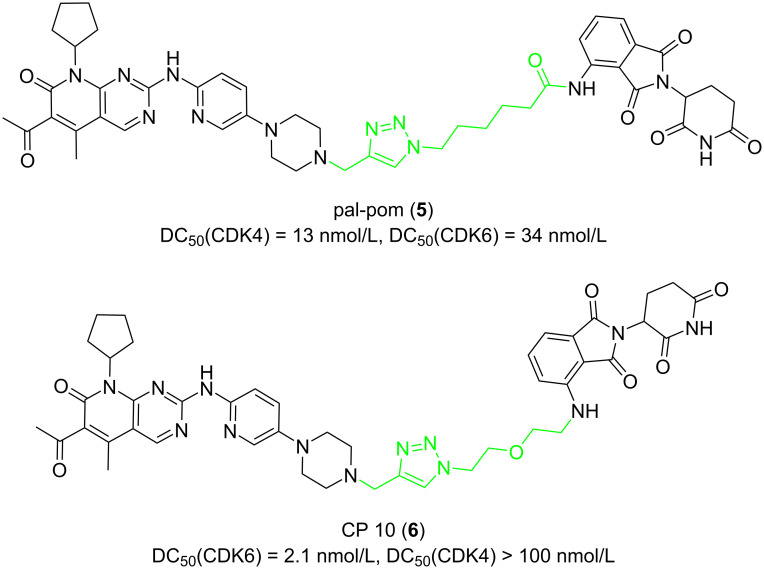
CDK4/6 PROTACs with triazole-containing linkers.

Currently, small-molecule inhibitors for the CDK family approved by the FDA all target CDK4/6. CDK4/6 has high homology and has been shown to play a crucial role in the development of breast cancer [26]. In recent years, it has been proved that expression of CDK6, but not of the highly related CDK4, is required for the proliferation and survival of Ph+ acute lymphoblastic leukemia cells. According to this, some researchers consider that CDK6 may be a more attractive target than CDK4 in some diseases [55]. Therefore, how to distinguish between CDK4 and CDK6, to achieve better treatment effects for certain specific diseases, such as Ph+ALL, has become a problem of concern to many pharmaceutical chemists. In 2019, Gray, Zhang and co-workers designed a PROTAC-1 (**1**) to degrade CDK4/6 [21]. To construct compound **1** they used the CDK4/6 inhibitor palbociclib as the POI ligand, pomalidomide, which combines with CRBN as the E3 ligand and a 4-carbon alkyl linker to connect them. The compound can simultaneously degrade CDK4 (the degradation rate exceeds 50% at 0.1 μM in Jurkat cells) and CDK6 (the degradation rate exceeds 95% at 0.1 μM in Jurkat cells), and it shows a certain degree of selectivity towards CDK6. In the same year, Gray, Winter et al. developed another PROTAC named BSJ-03-123 (**2**), which can selectively degrade CDK6 [56]. Compared to compound **1**, the POI ligand and the E3 ubiquitin ligase combined with the E3 ligand of compound **2** have not changed. The only difference is that the linker of compound **2** is longer and comprises a PEG chain (Figure 2). The results showed that compound **2** has a better ability to target the degradation of CDK6, and proteomics results also show that compound **2** has no degradation effect on CDK4. In 2020, Benowitz and co-workers carried out a project to explore the impact of different E3 ligands on the selective degradation of CDK4/6 [57]. In their research, they designed various PROTACs consisting of different linkers and E3 ligands, and compound **3** (Figure 3) is one of them.

Interestingly, when comparing compounds **3** and **2**, the POI ligand, the binding E3 ubiquitin ligase and the linker are identical with the only difference being the direction the linker connects the POI ligand and E3 ligand. Similarly, the research results show that compound **3** can also selectively degrade CDK6. Meanwhile, Rana, Natarajan and co-workers developed a PROTAC-6 (**4**) based on palbociclib and pomalidomide [58] (Figure 2). In the study, the researchers evaluated the degradation effect of compound **4** in HPNE and MiaPaCa2 cells, respectively. It was found that compound **4** could selectively degrade CDK6 without affecting other members of the CDK family (CDK2, 4, 5, 7, and 9).

Based on the above studies, it seems an effective development strategy to use a longer linker to connect the POI ligand and E3 ligand when designing PROTACs with high selectivity to target the degradation of CDK6. However, some studies did not support this hypothesis. When considering the influence of the linker on PROTAC selectivity, attention must be paid to the composition of the linker in addition to its length.

In 2019, Burgess and Zhao synthesized various different PROTACs, based on palbociclib and ribociclib as POI ligands, pomalidomide as E3 ligand, and linkers with triazole fragments to connect them [59] (Figure 3). The results showed that the PROTAC-pal-pom (**5**), which used palbociclib as the POI ligand, could induce the degradation of CDK4 and CDK6 in MDA-MB-231 cells. Its DC_50_ (concentration of 50% protein degradation) was 13 and 34 nmol/L, respectively. That is, compound **5** has a more pronounced ability to degrade CDK4 than CDK6. The same year, Rao, Wu et al. published their work on the design of PROTACs for the selective degradation of CDK6 [60]. They also constructed a PROTAC-CP 10 (**6**) containing a triazole-containing linker to connect palbociclib and pomalidomide. Compound **6**, a selective CDK6 degrader, showed good selectivity in U251 cells (DC_50_(CDK6) = 2.1 nM; DC_50_(CDK4) > 100 nM) and the compound did not degrade CDK1, 2, 5, 9. Analyzing these two studies, we can notice that when the linker used contains triazole fragments, maybe its length does not need to be as long as the linker, which contains a flexible chain to increase the selectivity for CDK6. On the contrary, shorter linkers seem more conducive to the selective degradation of CDK6.

In addition to CDK4/6, CDK9 is another family member that is getting a lot of attention. Many studies have shown that CDK9 is closely related to many types of cancer and it is crucial to cancer cells’ maintenance, growth, metastasis, and chemical resistance [61]. Although many small-molecule inhibitors have also been developed for CDK9 so far, due to the high homology of the CDK family, inhibitors always tend to target a variety of CDK members, thereby reducing their specificity as therapeutic agents and chemical probes and results in many adverse effects [62–63]. Based on such background, to target CDK9 with high selectivity to treat diseases related to CDK9 abnormal levels, PROTACs gradually came into view and began to be widely concerned and studied.

**Figure 4:**
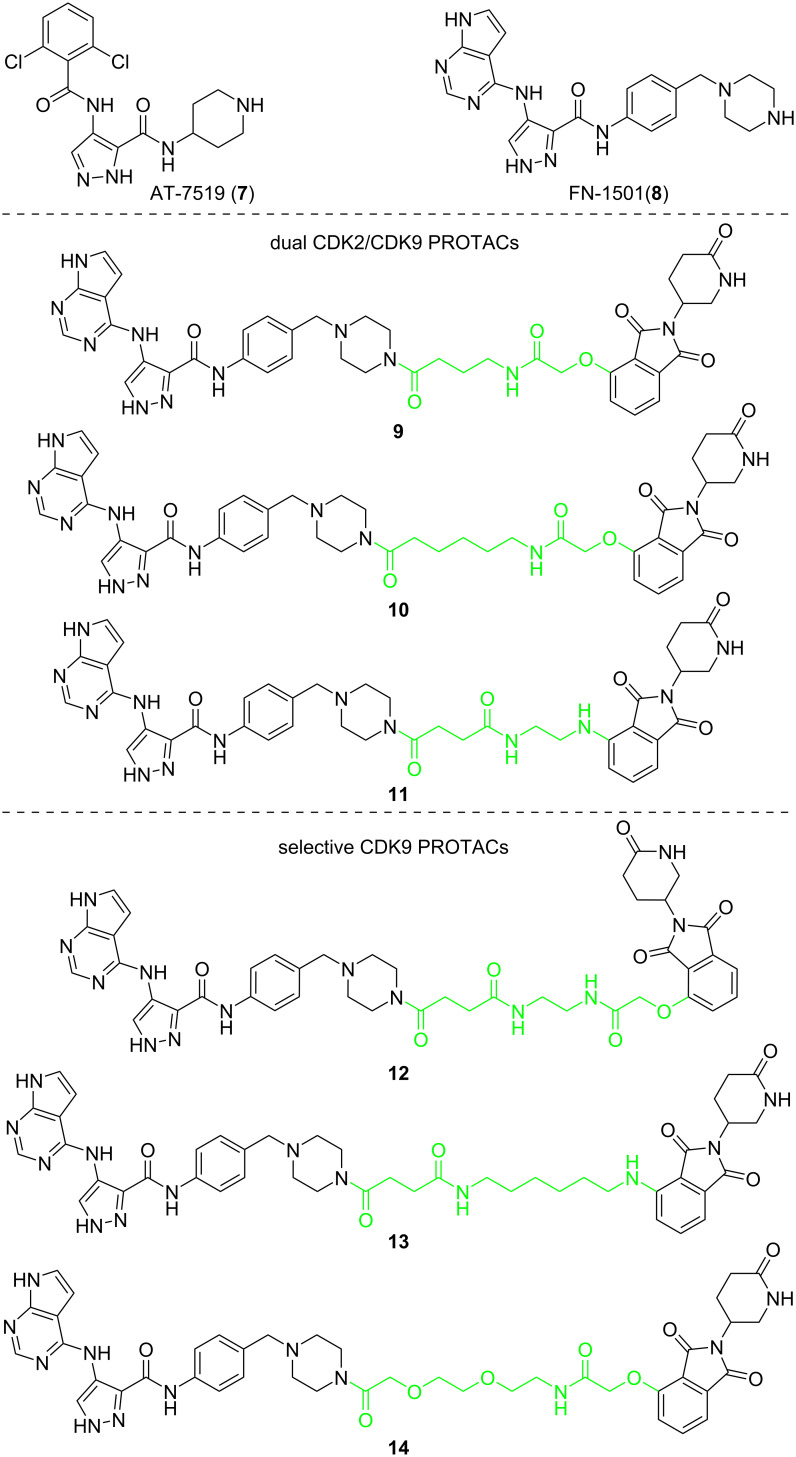
Structures of AT-7519 (**7**) and FN-1501 (**8**), and CDK9 degrading PROTACs based on compound **8** with varied lengths of linkers.

In 2020, Chen et al. found that the length of the linker will significantly affect the selectivity of PROTACs to CDK9 [64]. They used pan-CDK inhibitors AT-7519 (**7**) and FN-1501 (**8**) as POI ligands and connected them with CRBN ligands through various linkers. First, the results showed that PROTACs with molecule **7** as POI ligand did not achieve a good degradation effect on CDK9, whereas most PROTACs with molecule **8** as POI ligands showed an excellent ability to degrade CDK9 (**9**–**14**, Figure 4). More importantly, as for the PROTACs with molecule **8** as the POI ligand, linkers containing 8–10 atoms (**9**–**11**) resulted in CDK2/9 dual degraders. When the linker length expands to 11 or 12 atoms (compounds **12**–**14**), the CDK2/9 dual degraders become a selective CDK9 degrader.

**Figure 5:**
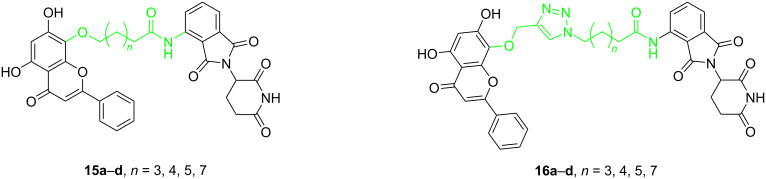
CDK9 PROTACs with alkane chain as linkers or triazole linkers.

Similar to the design of PROTACs for the targeted degradation of CDK6, in addition to the length of linkers, the composition also greatly influences selectivity. In 2018, Bian, Li and co-workers developed PROTACs based on wogonin to degrade CDK9 selectively [65]. In their research, they designed two types of linkers to connect the POI ligand and the E3 ligand. One type is based on alkane chains (Figure 5), and the other additionally includes a triazole fragment (Figure 5). By varying the length of the linker, they synthesized 8 compounds in total. The results showed that PROTACs containing triazole fragments (**16a**–**d**) were more effective in degradation than PROTACs comprising alkane chains only (**15a**–**d**) and they showed higher selectivity towards CDK9 between CDK9 and CDK5. The 10-atoms linker in **16c** showed the best CDK9 degradation activity among these compounds and the selectivity and degradation effect decreased when the linker length was more than 10 atoms.

#### HDAC

**Figure 6:**
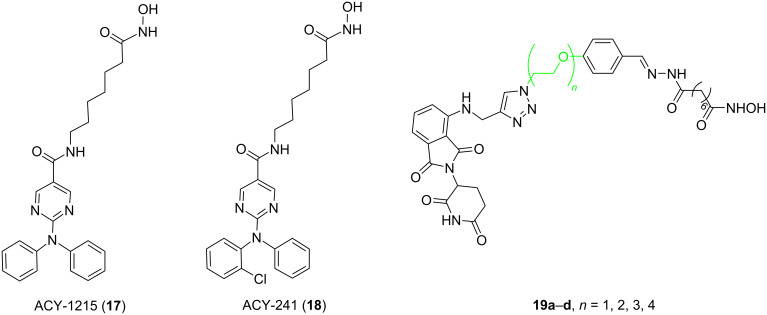
Structures of HDAC6 inhibitors ACY1215 (**17**) and ACY-241 (**18**), as well as the structure of PROTACs **19a**–**d** synthesized to target HDAC6 degradation.

Epigenetics plays a vital role in the occurrence, development, and metastasis of cancer [66–68]. The epigenetics process mainly includes DNA methylation, histone acetylation, histone methylation, and histone phosphorylation [69–71]. Histone acetylation is the most common type, and it is important for regulating the physiological process of normal cells. This process is controlled by histone deacetylases (HDACs) and histone acetyltransferases (HATs) [72]. Now, researchers have found 18 kinds of HDACs in total. They are divided into four categories: HDAC1, 2, 3, and 8 for class I, HDAC4, 5, 7, and 9 for class IIa, HDAC6 and 10 for class IIb, seven sirtuin proteins for class III, and HDAC11 for class IV [73]. Studies have confirmed that the expression and activity of HDACs are closely related to the occurrence of many types of cancers [74–76]. Abnormal expression of HDACs will significantly affect the occurrence, development, and migration of cancer [77–80]. Although an inhibition of HDACs by inhibitors can induce apoptosis and restrain cancer occurrence, the effectiveness of developing HDAC inhibitors to treat cancer is limited because these inhibitors often lack specificity and have toxic side effects [66,81–82]. For example, HDAC6, a class IIB HDAC isoenzyme, is involved in several signaling pathways associated with multiple neurological disorders, various cancers, and immune diseases [83]. A great deal of effort has been devoted to developing HDAC6 inhibitors. However, so far, only two compounds, ACY-1215 (ricolinostat, **17**) and ACY-241 (citarinostat, **18**) have reached clinical trials (Figure 6) [83]. Research has revealed that a variety of HDAC6 inhibitors are not suitable to effectively treat diseases and enter the clinical trials due to a lack of selectivity, which can cause serious side effects [84]. Consequently, developing drugs targeting HDACs specifically to treat cancer is urgent. In this context, PROTACs have become a straw to clutch to target HDACs specifically. In recent years, the design of various PROTACs targeting the HDAC family has become a hotspot. Besides, using what kind of linker to connect the POI ligand and the E3 ligand to improve the selectivity of PROTACs has also become the core problem in designing such molecules.

In 2018, Tang et al. developed the first PROTAC targeting HDAC6 degradation [85]. The study used a pan-HDAC inhibitor as the POI ligand and thalidomide as the E3 ligand to design four PROTACs (Figure 6, **19a**–**d**). These used linkers in these PROTACs all contained triazole fragments, but their lengths differed. Having synthesized the PROTACs, the authors explored the effect of linker length on the selectivity. The results showed that when the linker contained three ethyleneoxy fragments (**19c**), the resulting PROTAC demonstrated the best selective degradation effect on HDAC6 (by detecting the expression level of HDAC1 and HDAC2 as typical examples of class I HDACs, and HDAC4 and HDAC6 as typical examples of class II HDACs). The selectivity of PROTACs with other linker lengths (**19a**, **19b**, **19d**) was not as good as that of compound **19c**.

**Figure 7:**
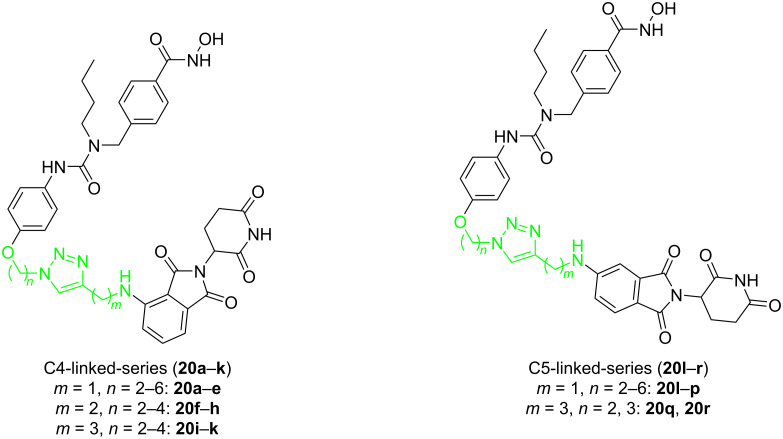
C4-linked-series and C5-linked-series of HDAC6 PROTACs.

In 2019, Tang et al. continued on designing PROTACs based on CRBN for selective HDAC6 degradation [22]. They used the selective inhibitor nexturastat A of HDAC6 with the CRBN E3 ligand pomalidomide to design 18 PROTACs. According to the different amino sites on the phthalimide ring of pomalidomide, these PROTACs were divided into two series: a C4-linker series and a C5-linker series (Figure 7). As for these compounds, the difference is the number of carbon atoms (*n*) between nexturastat A and the triazole fragment or the number of carbon atoms (*m*) between pomalidomide and the triazole fragment.

It was found that compound **20d** (DC_50_ = 1.64 ± 0.24 nM) with a medium-length linker (*n* = 5, *m* = 1) within the C4-linker series achieved the most potent selective degradation (compared to HDAC1, 3, 4) in the subseries **20a**–**e** (*m* = 1, *n* = 2–6). For the subseries **20f**–**h** (*m* = 2, *n* = 2–4), when the linker length increased, the molecular degradation efficiency increases, and **20h** (*n* = 4, *m* = 2) was the best (at 100 nM degradation was 78.3–80.1% of HDAC6). In the subseries **20i**–**k** (*m* = 3, *n* = 2–4), compound **20i** with the shortest linker (*n* = 2, *m* = 3) has been the most effective degrader (at 100 nM degraded 82.1–84.1% of HDAC6) (Figure 7). In the C5-linker series, the selective degradation of the **20l**–**p** (*m* = 1, *n* = 2–6) series increased with the increase of linker length. Compounds **20o** (*n* = 5, *m* = 1) and **20p** (*n* = 6, *m* = 1) demonstrated similar ability to degrade HDAC6 (**20o** at 100 nM degraded 83.7–85.7% of HDAC6 and **20p** at 100 nM degraded 83.0–84.0% of HDAC6). Members of the subseries **20q** and **20r** (*m* = 3, *n* = 2 and 3) showed relatively low effects on HDAC6 degradation. These results showed that the optimal total number of methylene units in the linker is about 6, and the C4-linker series is slightly stronger than the C5-linker series. Therefore, the difference in degradation selectivity may be related to the ubiquitination sites available to HDAC6 by E3 ligase recruited by the degrader and both, distance and connection sites maybe affect it.

**Figure 8:**
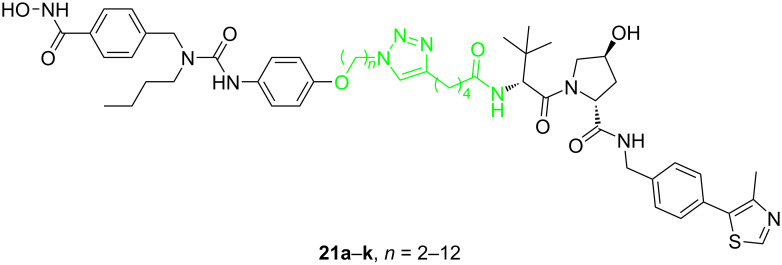
Structures of VHL-based degraders.

In 2020, Tang et al. continued with reporting studies on the development of PROTACs for the selective degradation of HDAC6 [86]. This time they used VHL as the E3 ubiquitin ligase. They also designed several series of compounds and used linkers with different types and lengths to connect the POI ligand and VHL ligand. Among them, compounds **21a**–**k** showed good activity. The number of methylene units (*n*) between the POI ligand and the triazole fragment ranged from 2 to 12, and there were four methylene units between the triazole ring and the VHL ligand. So these compounds were called the “*n* + 4” series. Although they also tested the "*n* + 2", "*n* + 3" series of compounds, and the "*n* + 4" series of compounds with the ethyleneoxy unit instead of the methylene unit, they found that these PROTACs were usually not active degraders, so no further research in this direction was carried out. When testing all 11 "*n* + 4" VHL-based potential degraders, it was found that the molecular selectivity almost steadily increased with the linker length starting from compound **21a** (*n* = 2) to **21k** (*n* = 12) (Figure 8). Among them, compound **21j** showed the best selectivity and degradation effect of HDAC6 compared to HDAC1, 2, 3, 4, 7, 8 (DC_50_ = 7.1 nM). A comparison of the above two studies, one can find that the linker length required by degraders based on the VHL ligand is much longer than that of PROTACs based on the CRBN ligand. Thus, it is evident that the linker's effect on PROTAC selectivity is not independent, and it is often closely related to the E3 ligand and even the POI ligand.

**Figure 9:**
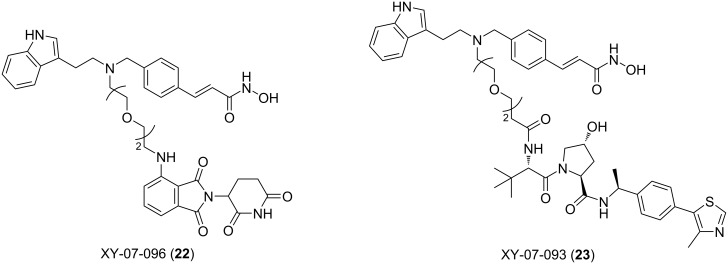
Structures of VHL-based and CRBN-based selective HDAC PROTACs.

In 2021, Fischer and colleagues provided a detailed study on PROTACs for selectively targeting the HDAC protein family [87]. Some previous studies suggested that modifying the length of the linker is an effective strategy to change the selectivity of degraders, which was also confirmed in the study by Fischer et al. [23,43,88]. To systematically explore the effect of the linker length on HDAC degradation selectivity, they synthesized two series of multitarget degraders based on dacinostat that can recruit different E3 ubiquitin ligases (CRBN and VHL). In both series, they designed molecules containing 1–5 ethyleneoxy units as the linker. Then they compared the differences of each series to reveal the key impact caused by the change in linker length. Remarkably, for PROTACs that recruit VHL XY-07-093 (**23**, Figure 9), using shorter or longer linkers can afford PROTACs that selectively degrade HDAC3. However, when the linker contains 4 ethyleneoxy fragments, HDAC degradation activity is completely lost. This is surprising because compounds with similar linker lengths (containing 3 ethyleneoxy fragments and 5 ethyleneoxy fragments) are effective and active degraders. Similarly, when using CRBN as the E3 ligand, they designed the PROTAC molecule XY-07-096 (**22**). The degradation activity of HDAC is completely lost with the increase of the linker (containing 5 ethyleneoxy fragments). In contrast, the PROTAC with a shorter linker is a molecule that can selectively degrade HDAC6 and HDAC8.

#### p38 MAPK

The p38 mitogen-activated protein kinase (MAPK) family comprises four members: p38α (MAPK14), p38β (MAPK11), p38γ (MAPK12), and p38δ (MAPK13), with p38α being widely expressed and the most abundant member in almost all cells [89]. There is a high degree of homology among the p38MAPK family members with p38α and p38β having 75% homology, p38γ and p38δ having 62% and 61% homology with p38α, respectively, and p38γ and p38δ having 70% homology [90–92]. The p38MAPK family has been widely studied as a potential therapeutic target for the past two decades. Many studies have shown that p38α, p38β, p38γ, and p38δ all play a crucial role in cellular processes related to cancer and inflammatory diseases [93–96]. So developing corresponding small-molecule drugs with the p38MAPK family as a key target is necessary. However, up to now, no small-molecule inhibitors targeting the p38MAPK family have been approved by the FDA, since every effort has failed in clinical trials [97–99]. The reasons for the failure are rarely discussed but one of the factors is believed to be due to the inhibition of several p38MAPK proteins [98]. Therefore, how to specifically target p38MAPK family members has become a vital issue in the development of drugs. In this regard, PROTAC again shows its unique advantages in selectivity for highly homologous proteins compared with small-molecule inhibitors [100].

**Figure 10:**
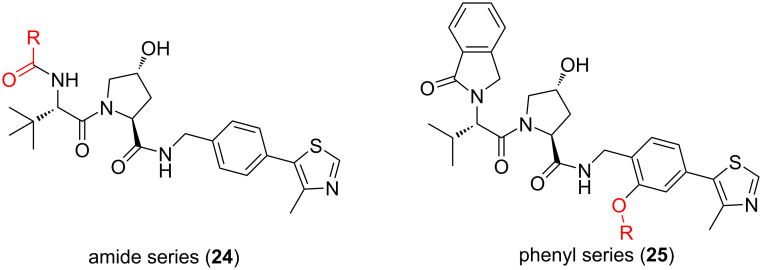
Structures of the “amide series” and “phenyl series” PROTACs studied by Crews et al.

In 2019, Crews et al. developed PROTACs, able to specifically degrade p38α and p38δ [23]. They used foretinib as the POI ligand and two E3 ligands with different structures to target VHL to explore the influence of the direction of VHL recruitment of PROTACs on selectivity (termed the “amide series” and “phenyl series” individually). Simultaneously, they used four linkers of different lengths to connect the E3 ligand and the POI ligand to explore the influence of linker fragments on molecular selectivity (Figure 10).

**Figure 11:**
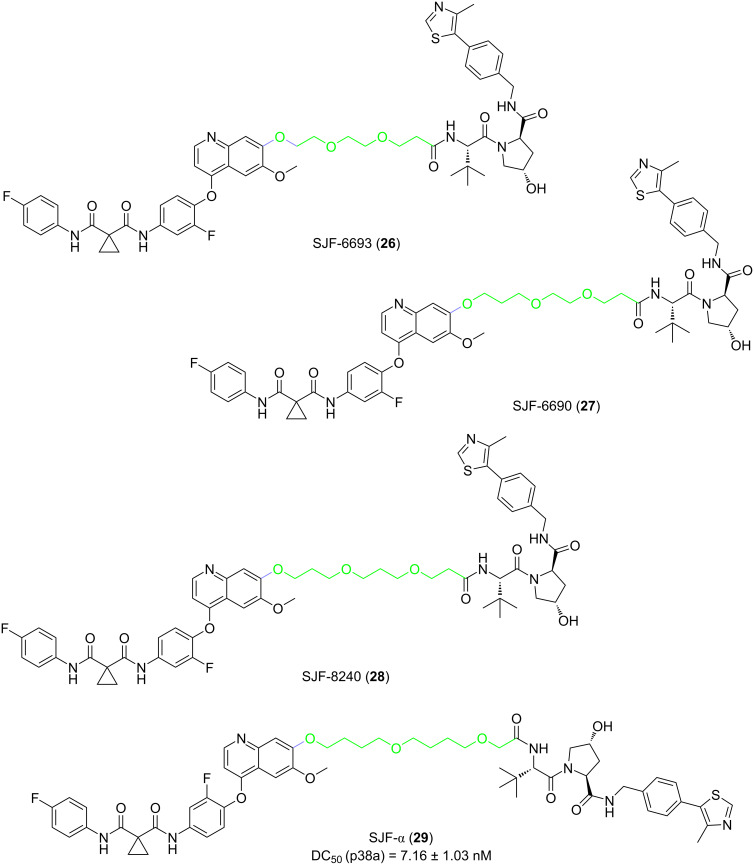
Structures of the “amide series” PROTACs.

As for the “amide series”, the authors discovered that in MDA-MB-231 human breast cancer cells, the 10-atom and 11-atom linker PROTACs (SJF-6693 (**26**) and SJF-6690 (**27**)) were not selective and degraded both p38α and p38δ nonspecifically with DC_50_ < 100 nM (Figure 11). However, the 12-atom and 13-atom linker PROTACs SJF-8240 (**28**) and SJF-α (**29**) can selectively degrade p38α (Figure 11). Compound **29** degraded p38α with a DC_50_ of 7.16 ± 1.03 nM and maximum degradation, D_max_ of 97.4%, but the effect on the degradation of p38δ was much worse (D_max_ = 18%, DC_50_ = 299 nM). Other p38 isoforms (β and γ) were not degraded when the concentration was up to 2.5 µM.

**Figure 12:**
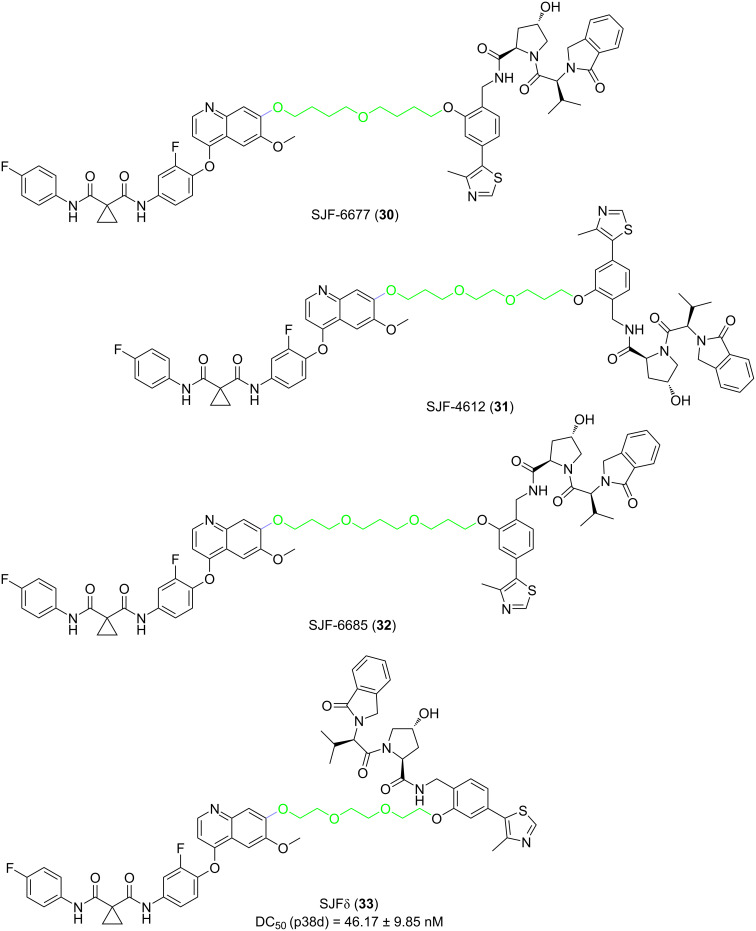
Structures of the “phenyl series” PROTACs.

On the contrary, PROTACs with the long linker in the "phenyl series" showed almost no ability to degrade the p38 subtype (**30**, **31**, **32**, **33**, Figure 12). When the linker length in the "phenyl series" was reduced to 10 atoms, the resulting PROTAC showed a robust selective degradation of p38δ. Compound **33** degraded p38δ with a DC_50_ of 46.17 ± 9.85 nM and D_max_ of 99.41 ± 3.31% but did not degrade α, β, or γ at all.

In some cases, even changing single carbon atoms of the linker segment may cause a surprising change in the degradation selectivity of PROTACs. For example, compound **28** shows submicromolar degradation of two p38 subtypes. Once the linker contains an additional carbon atom, the resulting PROTAC **29** degrades p38α at a nanomolar concentration and p38δ at a micromolar concentration. Similarly, the 10-atom-linker-containing compound **33** can almost completely degrade p38δ, but in the degradation of p38α, it is restricted. In contrast, the structure of compound **30** having additional carbon atoms added to the linker, resulted in less than 50% degradation of both subtypes at the maximum efficiency. It can be seen that the length of the linker is crucial for the selective degradation of PROTACs. The proper linker length enables PROTACs to distinguish protein families with more than 60% homology. Nevertheless, this characteristic is unimaginable for small-molecule inhibitors.

#### BET

Bromodomain and extraterminal domain (BET) proteins are epigenetic readers. They comprise the ubiquitously expressed BRD2, BRD3, and BRD4 and the testicular-specific expressed BRDT [101]. The function of the BET protein family is mainly to regulate gene transcription by recognizing acetylated lysine residues on histones [102–103]. The imbalance of BET protein activity, especially BRD4, is closely related to cancer and inflammatory diseases, so the BET protein family has become an attractive drug target [102]. Because BET proteins play an essential role in various diseases, many small-molecule inhibitors against BET have been developed [104]. However, the clinical studies for the BET inhibitors were not successful because the compounds did not show good antitumor activity [105]. One possible reason for the failure of these trials is that most of the BET inhibitors that entered the clinical trials are pan-BET inhibitors so they can bind multiple BET protein members [106]. However, due to the varying expression levels of BET protein members in different tumor types, these inhibitors failed in displaying good therapeutic effects. Moreover, due to the lack of highly selective BET inhibitors, the mechanism of cancer signaling pathways related to BET proteins is not yet clear. Therefore, it is necessary to develop drugs with high selectivity towards specific BET members.

**Figure 13:**
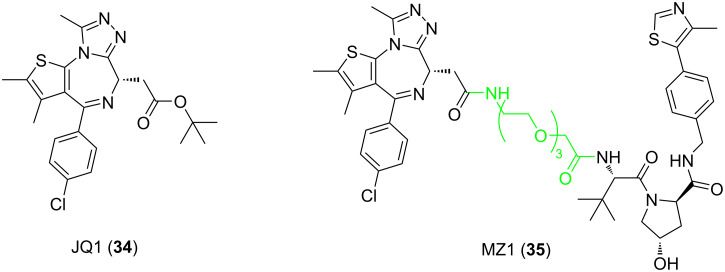
Structures of JQ1 (**34**) and MZ1 (**35**).

In order to solve this problem, Ciulli et al. developed a molecule that selectively degrades BRD4 using the PROTAC technology in 2015 [107]. They used JQ1 (**34**), a non-selective BET protein family inhibitor, as the POI ligand and selected linkers of different lengths composed of PEG chains with three or four ethyleneoxy units to connect **34** with the VHL ligand, thus designing four PROTACs. The results showed that MZ1 (**35**, Figure 13), whose linker is composed of three ethyleneoxy units in the structure, has an excellent ability to degrade BRD4 selectively (compared to BRD2 and BRD3). This indicates that the linker affects the selectivity of PROTACs to degrade BET protein family members. The evidence that more directly proves this view comes from another study reported by Ciulli et al. in 2017 [41]. In this study, they analyzed the ternary composite structure of PROTACs for the first time and explained the selectivity of compound **35** in detail.

**Figure 14:**
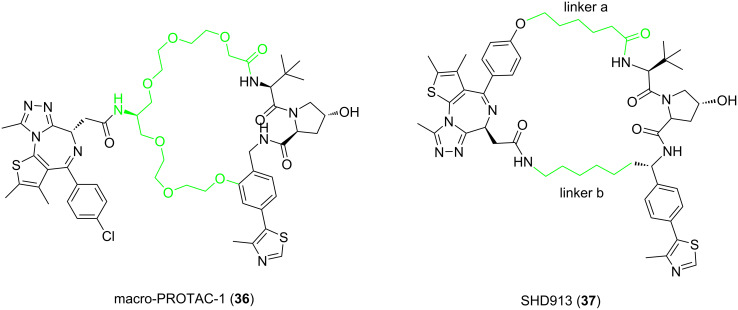
Structures of **macro-PROTAC-1** and **SHD913**.

In 2020, Ciulli et al. continued to develop a PROTAC with a large ring linker (macro-PROTAC-1, **36**, Figure 14) based on their previous research [108]. This PROTAC also selectively degrades BRD4, whereas BRD2 and BRD3 can only be degraded at high concentrations. By comparing the structures of the two molecules **35** (Figure 13) and **36**, it becomes clear, that the main difference between them is that in compound **36** the POI ligand and VHL ligand are connected via a conformation-restricting macrocyclic linker. Although this seems to be a minor change, the comparison of these two studies reveals that compound **36** is able to cooperatively form ternary complexes with the BD2 domains of BRD4, BRD2, and BRD3. The cooperativity factor α, a factor reflecting effects of PPIs on ternary complex formation, was found to be αBRD2^BD2^ = 10.5, αBRD3^BD2^ = 9.5, and αBRD4^BD2^ = 4.0. Meanwhile, the study did not observe a synergy between compound **36** and the first bromodomain (BD1) of BRD2, BRD3, and BRD4 (αBRD2^BD2^ = 0.7, αBRD3^BD2^ = 0.9, αBRD4^BD2^ = 0.8). This result is in sharp contrast to compound **35**, which forms synergistic complexes with all bromodomains of the BET protein family (αBRD2^BD1^ = 2.9, αBRD2^BD2^ = 2.3; αBRD3^BD1^ = 3.5, αBRD3^BD2^ = 10.7, αBRD4^BD1^ = 2.3, αBRD4^BD2^ = 17.6). These results are promising because all members of the BET protein family contain two highly homologous bromodomains (BD1 and BD2) with 49% homology [88,109]. From here, it is clear, that the change of linker fragments can not only play a decisive role in the differentiation of BET protein family subtypes but also allows distinguishing BD1 and BD2 domains in the different subtypes. This discovery shows that the PROTAC technology offers substantial advantages in terms of selectivity and that the design of suitable linkers for the application of this technology to the selective degradation of highly homologous protein families is of great significance.

In 2024, Ding et al. reported the "head-to-tail" macrocyclic PROTAC, SHD913 (**37**) [110]. Based on the crystal structure of the BRD4^BD2^:MZ1:VHL ternary complex, the authors designed two short-chain linkers for macrocyclization, targeting two critical distances (4.6 Å and 7.1 Å) between the warhead molecule **34** and the VH032 ligand. Through systematic optimization of linker lengths, it was found that the molecule achieved optimal activity when the linker a consisted of six methylene groups and linker b consisted of three methylene groups. The resulting products **37** exhibited DC_50_ values of 7.7 nM and 5.0 nM for the long and short isoforms of BRD4 in PC-3 cells, respectively (Figure 14). Regarding selectivity, compound **37** demonstrated a significant degradation advantage for BRD4 over other BET family members: Western blot analysis showed that BRD4 was effectively degraded at a 30 nM treatment concentration, while BRD2/3 remained largely unaffected. Global proteomic analysis confirmed that BRD4 was the most significantly downregulated protein; the downregulation of BRD3 was markedly lower, and no significant change was observed for BRD2. Furthermore, while the DC_50_ values for BRD4 were in the single-digit nanomolar range, the DC_50_ values for BRD2/3 were both well above 100 nM. Due to the conformational constraint imposed by the macrocyclic linker, compound **37** did not exhibit a "hook effect" even at concentrations as high as 60 μM, whereas the linear control molecule **35** showed a decline in activity at 30 μM. NanoBRET and ITC experiments indicated that compound **37** induces a stable protein–protein interaction interface between BRD4^BD2^ and VHL through linker-mediated conformational locking, achieving a cooperativity factor (α) of 117, which is significantly higher than those of compounds **35** and **36**. Its co-crystal structure revealed that linker b forms hydrophobic interactions with the ZA loop of BRD4^BD2^ and stabilizes the PPI interface via a conserved salt-bridge network, while linker a acts as a "hinge" to bring the two proteins into proximity. This study systematically elucidated how macrocyclic linker length and composition regulate PROTAC selectivity, providing a reference design strategy for the precise targeting of highly homologous protein families through linker engineering.

The above highlighted examples summarized how to use the PROTAC technology to achieve highly selective degradation for some selected highly homologous proteins by changing the linker. Based on the results, it can be concluded that for different highly homologous proteins, there is no fixed conclusion about what kind of linker can better improve the selective degradation ability. What’s more, it is difficult to answer whether more attention should be paid to the length of the linker or different linker compositions when designing PROTACs. Facing these problems, it is often needed to analyze, design, and explore on a case by case basis. Even, when using different E3 ligands or POI ligands to explore the influence of the linker fragments, completely opposite conclusions may be drawn. This indicates that although the linker is vital for the design of PROTACs, the impact of the POI ligand and E3 ligand need to be considered. PROTACs function as a whole, not just one or two parts and therefore, the POI ligands, linker, and E3 ligands cannot be analyzed separately. Of course, this does not mean that examinations and discussions of individual linkers are pointless. A detailed knowledge of the SAR of a linker fragment of PROTACs for different highly homologous protein families allows to quickly and specifically select the linker segment with the best selectivity and activity to synthesize PROTACs in the future. It also reduces the time required for exploration during the development of highly selective PROTACs. In addition, it can give full play to the remarkable selectivity of the PROTAC technology compared with small-molecule inhibitors.

### Influence of E3 ligands on the selectivity of PROTACs in highly homologous protein families

It is well known that PROTAC is a technology for the specific degradation of proteins through the ubiquitin-proteasome system [111]. Ubiquitination refers to the process of covalently binding ubiquitin to target proteins under the catalysis of a series of enzymes [112]. The ubiquitinated protein can be recognized explicitly by the proteasome to achieve degradation. The whole process requires the participation of three enzymes: ubiquitin-activating enzyme (E1), ubiquitin-conjugating enzymes (E2), and ubiquitin-ligase enzymes (E3) [113–115]. So far, two E1 enzymes, about 40 E2 enzymes, and more than 600 E3 enzymes have been found in the human proteome [116]. As the specificity of the substrate protein in the ubiquitination is determined by E3 ubiquitin ligase, the E3 enzyme is a colossal protein family compared with the E1 enzyme and E2 enzyme. However, although there are many kinds of E3 enzymes, the most targeted combination of E3 ligands when designing PROTACs are Von-Hippel-Lindau (VHL), cereblon (CRBN), "inhibitor of apoptosis" protein (IAP), and MDM2 [117]. Among these four E3 ubiquitin ligases, VHL and CRBN are the most commonly used E3 ubiquitin ligases when designing PROTACs. This is because VHL ligands and CRBN ligands often have several favorable characteristics: (1) speciﬁc, strong, biophysically validated binding afﬁnity for their target E3 ligases; (2) acceptable physicochemical characteristics such as molecular weight, solubility, lipophilicity, lack of metabolic hot spots; and (3) well characterized structural information of their binding modes [118–119].

**Figure 15:**
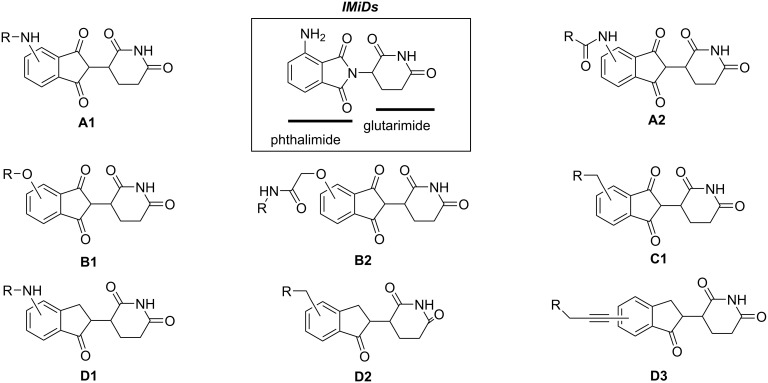
Commonly utilized thalidomide-derived CRBN ligands and possible linker attachment styles. **A1**, **A2**: pomalidomide derivatives; **B1**, **B2**: 4-hydroxythalidomide derivatives; **C1**: alkyl-type attachment to thalidomide; **D1**–**D3**: lenalidomide derivatives.

**Figure 16:**
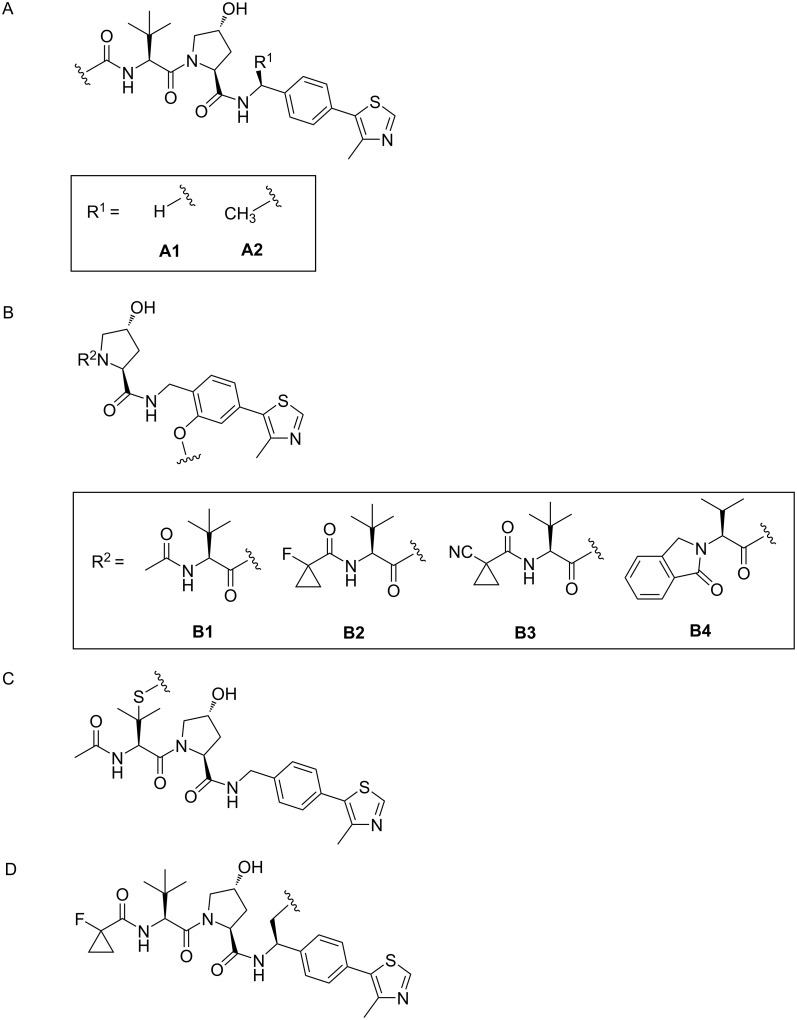
VHL ligands frequently used in PROTACs. Linker attachment options are represented with curly bonds and are: (A) via an amide bond after *tert*-leucine; (B) phenolic linkage point at the benzene ring; (C) via a thioether at the left-hand side amino acid; and (D) via the benzylic methylene group.

CRBN, one of the most commonly used E3 ligand to target E3 enzymes in PROTAC molecular design, has been successfully used to target and degrade more than 30 different proteins [119] (Figure 15). These proteins include proteins related to various cancers, immune diseases, and even neurodegenerative diseases such as Tau [120–122]. VHL (Figure 16), another E3 ubiquitin ligase frequently used in PROTAC molecular design, has also been successfully used to target and degrade more than 20 different proteins [123].

It is precisely because more and more E3 ligands are being developed that the design of PROTACs has become more rich and diverse. With the deepening of research, it is found that different E3 ligands in the structure of PROTACs containing the same POI ligand and linker fragments will also induce protein degradation at different levels [117]. What is more important, there will be some differences in the degradation selectivity of POI. Therefore, this part of the review focusses on the influence of different E3 ligands in PROTACs on the overall molecular degradation selectivity.

#### E3 ligands target different E3 ubiquitin ligases

During the design of PROTACs, the selected E3 ligands often target CRBN or VHL, however, in current research there is a preferential use of CRBN as E3 ubiquitin ligase instead of VHL. This is because ligands targeting CRBN have better drug-likeness properties, including lower molecular weight, fewer hydrogen bond donors in their structures, and fewer rotatable bonds [124]. However, in addition to influencing the physical and chemical properties of molecules, according to many relevant research results, it was found that when the selected E3 ubiquitin ligases are different, the selectivity of PROTACs indeed show significant differences. As a result, different E3 ligands targeting different E3 enzymes will have a vital impact on the selectivity of PROTACs in highly homologous protein families.

**BCR-ABL and c-ABL:** Currently, the vast majority of chronic myeloid leukemia and about 20–30% of acute lymphoblastic leukemia are caused by chromosome translocation between chromosomes 9 and 22 [125]. The gene rearrangement leads to the expression of the oncogenic fusion protein, BCR-ABL and the loss of autoinhibition of the c-ABL kinase domain in BCR-ABL is the main cause of cancer [126]. Imatinib mesylate was the first tyrosine kinase inhibitor (TKI) targeting BCR-ABL [127–128]. It can competitively bind to the ATP binding site of c-ABL to inhibit the functions of c-ABL and BCR-ABL, thereby inhibiting cell proliferation [125]. Because long-term treatment with imatinib will lead to drug resistance in CML patients, the second generation of TKI was subsequently developed for treatment [129]. Although TKIs targeting BCR-ABL have achieved significant therapeutic effects, CML patients often need to take such drugs for life to control the disease's further deterioration. Based on these circumstances, if the PROTAC technology can be used to target the degradation of the BCR-ABL protein, it may maintain a good therapeutic effect for CML and reduce the time for CML patients to take medicine. Therefore, to achieve good degradation of BCR-ABL by PROTACS has been the focus of intensive pharmaceutical chemistry research. Interestingly, it was found that using different POI ligands and E3 ligands combinations can achieve efficient selectivity for BCR-ABL and c-ABL. This discovery is of great significance for the subsequent development of PROTAC targeting other proteins with high homology.

**Figure 17:**
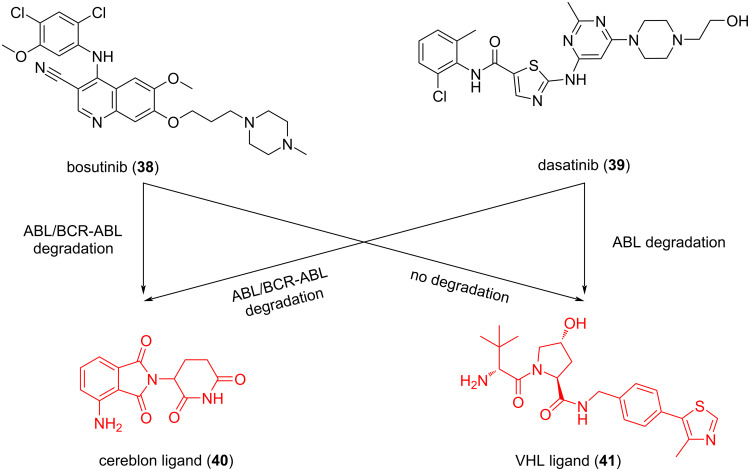
Varying the inhibitor warhead and the recruited E3 ligase permits targets to be accessed for degradation.

In 2016, Crews and colleagues designed different PROTACs by connecting BCR-ABL TKI dasatinib (**38**) and bosutinib (**39**), which target the binding of the c-ABL kinase domain, with cereblon (CRBN) ligand **40** or Von Hippel-Lindau (VHL) E3 ligand **41** through a linker (Figure 17) [126]. Interestingly, it was found that when molecule **38** was combined with pomalidomide to recruit CRBN, the resulting dasatinib-CRBN PROTAC not only retained its ability to induce c-ABL degradation (1 μM > 85%) but also induced BCR-ABL degradation (1 μM > 60%). However, when molecule **38** was combined with a VHL ligand to recruit VHL for degradation, the dasatinib-VHL PROTAC only degrades c-ABL and has no degradation effect on BCR-ABL. Meanwhile, the study also found that the PROTACs targeting VHL can still effectively bind and inhibit c-ABL and BCR-ABL in cell culture. This shows that the failure of these PROTACs to achieve degradation cannot be attributed to the loss of binding affinity. In addition, when molecule **39** is combined with pomalidomide to recruit CRBN, the formed PROTACs can degrade c-ABL and BCR-ABL. However, when molecule **39** is combined with a VHL ligand to recruit VHL for degradation, the bosutinib-VHL PROTAC does not display any degradation function. The authors believed that the proximity between the E3 ubiquitin ligase and the target protein is important for specific lysine residues required for ubiquitination degradation. They speculated that to achieve the selective degradation of BCR-ABL, specific E3 ligands, linkers, and POI ligands are needed when designing PROTACs to make POI and E3 ubiquitin ligases spatially close to each other to achieve ubiquitination. Thus, although many of the current PROTAC optimizations focus on linker fragments, the E3 ligands are also crucial for PROTACs to play an overall role in drug efficacy and selectivity.

**CDK:** CDK is a protein family with many highly homologous members. In recent years, PROTAC technology has been widely used in the specific degradation of CDK proteins to treat related diseases. What’s more, as mentioned above, achieving selectivity in the CDK protein family is crucial for certain diseases, thus some studies have explored the influence of E3 ligands differences on the selective degradation of CDK protein family members.

In 2020, Benowitz et al. designed a series of PROTACs, including VHL ligands, CRBN ligands, and IAP ligands, to explore the difference between the E3 ligands of PROTACs for the selective degradation of CDK4 and CDK6 (Table 1) [57]. The results showed that PROTACs based on CRBN, VHL, and IAP ligands can degrade CDK4 and CDK6 and show a specific selectivity for CDK6. Moreover, the PROTACs using CRBN ligands have better selectivity, while the PROTACs using VHL and IAP ligands display poor selectivity.

**Table 1:** CDK4/6 PROTACs based on VHL, IAP, and CRBN ligands.

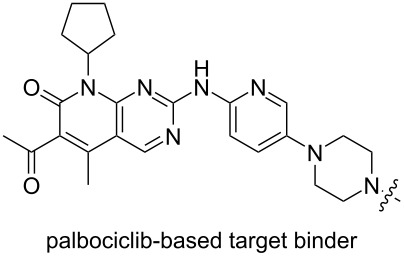			

ligand	E3 ligase ligand VHL	E3 ligase ligand IAP	E3 ligase ligand cereblon (CRBN)
	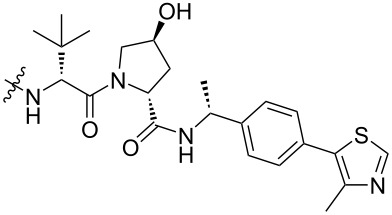 VHL	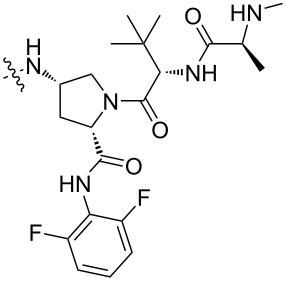 IAP	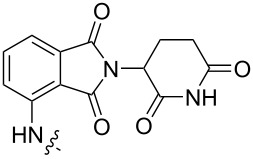 CRBN

linker	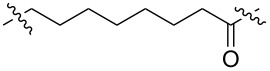	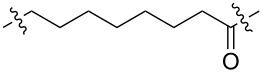	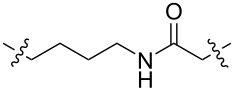
linker	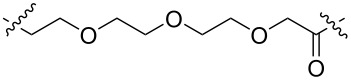	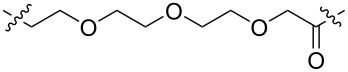	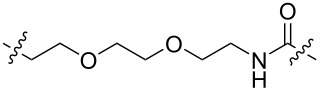
linker	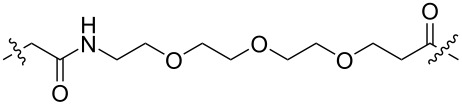	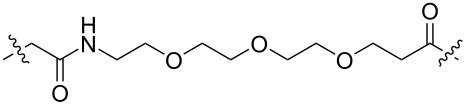	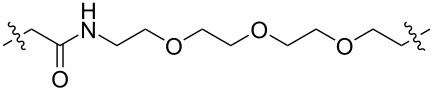

This conclusion has also been demonstrated by Wu, Rao et al. [60]. They found that PROTACs designed with other E3 ligands (VHL, cIAP, and MDM2) except CRBN could not effectively degrade CDK6 at 1 μM. In contrast, PROTACs designed with CRBN ligands were effective degraders of CDK6. However, unfortunately, only such a phenomenon was mentioned in their study and no detailed research or discussion was conducted. We look forward to conducting more relevant research on this interesting phenomenon in the future.

**Figure 18:**
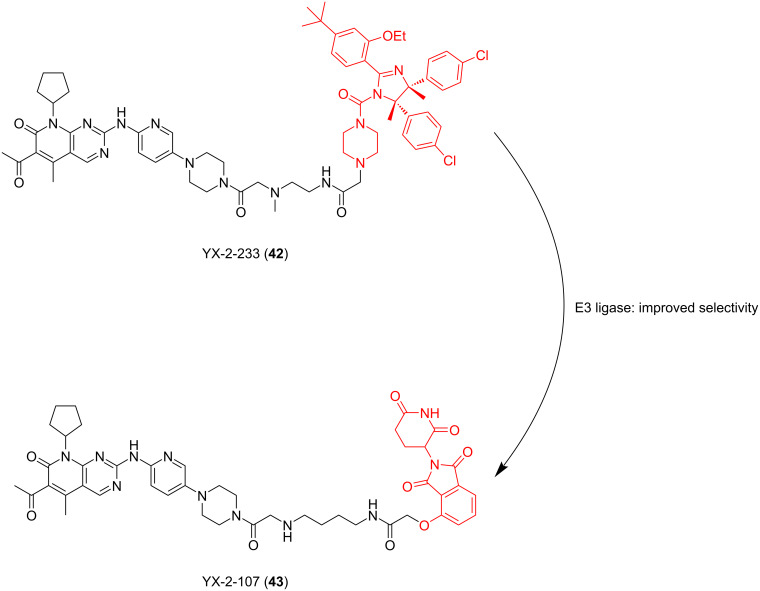
Structures of YX-2-233 (**42**) and YX-2-107 (**43**).

In the same year, Calabretta, Salvino and co-workers also reported their work on developing PROTACs for the selective degradation of CDK6 [130]. In order to treat Philadelphia chromosome-positive acute lymphoblastic leukemia (Ph^+^ALL), they designed a series of PROTACs based on different E3 ligands and linkers. The results showed that PROTAC YX-2-107 (**43**), which recruited CRBN to degrade CDK6, had the best effect (DC_50_ = 4 nM, IC_50_ = 4.4 nM in BV173 cells). Compared with YX-2-107, YX-2-233 (**42**) is structurally different from compound **43** only in the E3 ligand fragment. The E3 ligand used in **42** is MDM2 (Figure 18). The results showed that the degradation ability of compound **42** decreased significantly, and it became a CDK4/6 dual degrader, which did not show high selectivity for CDK6. It can be seen that the selection of appropriate E3 ligand fragments may not only result in good selectivity for highly homologous target proteins but also effectively enhance the degradation effect of PROTACs.

**Figure 19:**
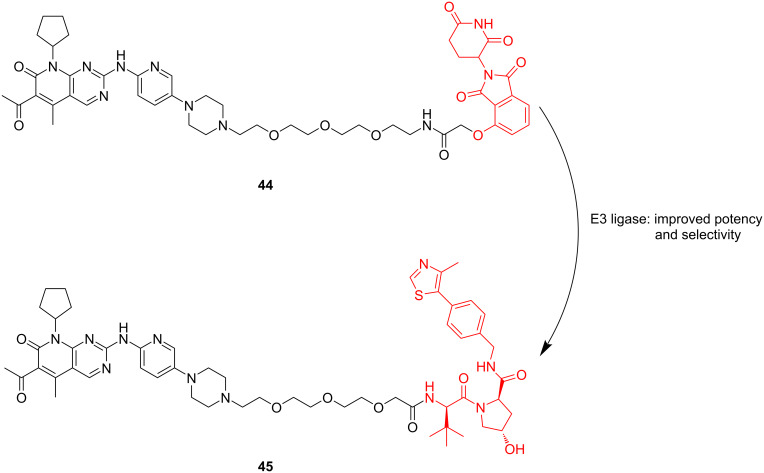
Structures of compounds **44** and **45**.

Based on the above results, CRBN ligands seem to be a better choice when developing highly selective PROTACs targeting CDK6 degradation. However, in 2020, Krönke, Gütschow and colleagues reported a study denying this conclusion [131]. They selected compound **44** as the lead compound and replaced the E3 ligand CRBN with VHL to synthesize compound **45**. The compounds were tested in MM.1s cells to evaluate their activity. The results showed that although the degradation efficiency of compound **45** was close to that of compound **44**, compound **45** showed significant advantages in selectivity for CDK4 and CDK6 (compound **45**: DC_50 for CDK4_/DC_50 for CDK6 (at 0.1 μM)_ = 19; compound **44**: DC_50 for CDK4_/DC_50 for CDK6 (at 0.1 μ M)_ = 4.9). Form these results, it can be seen that when designing PROTACs for different highly homologous target proteins, it is often necessary to consider the whole molecule rather than only one segment (Figure 19). Maybe when different linkers are used to connect the POI and E3 ligands, the difference in the E3 ligands may lead to the opposite result of selectivity.

**Figure 20:**
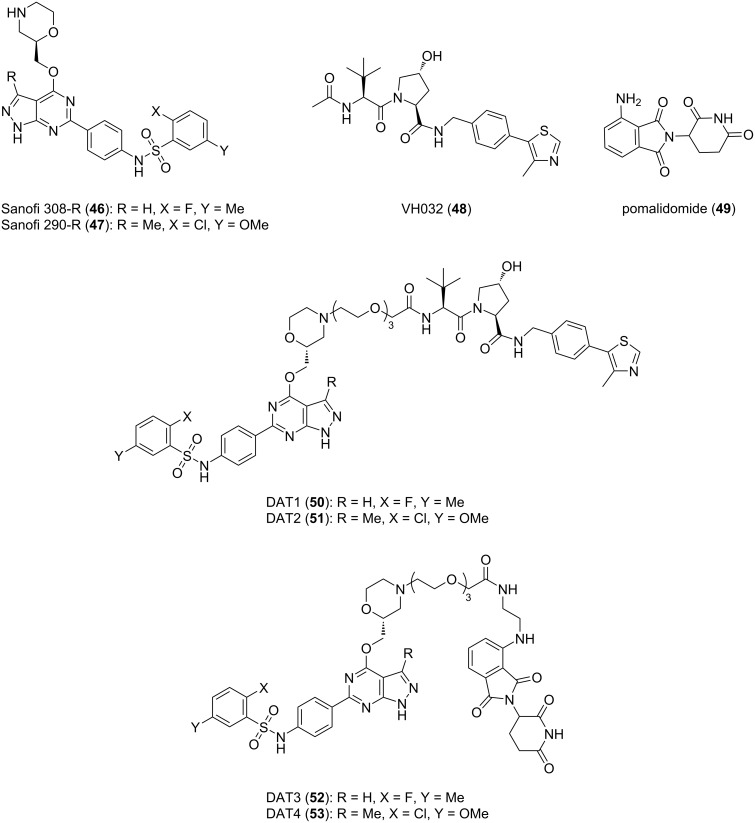
Design of the SGK3 PROTACs.

**SGK:** Serum-glucocorticoid-induced protein kinase (SGK) plays a key role in mediating resistance to phosphoinositide 3-kinase (PI3K)/Akt inhibition in breast cancer cells [132]. It has been reported that different ATP competitive inhibitors have similar affinity for all SGK isoforms [133–134]. Due to the high homology and structural similarity of catalytic domains between different SGK subtypes, no specific inhibitors have been developed [135]. To cope with this challenge and to better treat corresponding diseases, Alessi, Ciulli et al. used the PROTAC technology to develop effective and highly selective SGK3-PROTACs [136] (Figure 20). In the study, they first designed two types of POI ligands, Sanofi 308-R (**46**) and Sanofi 290-R (**47**), which can inhibit the kinase activity of SGK3. However, they lack specificity and have a particular inhibitory effect on S6K1. Then they connected the two POI ligands to VHL ligand VH032 (**48**) and cereblon ligand pomalidomide (**49**) through a medium-length linker composed of three ethyleneoxy units to form PROTACs. The results showed that only DAT1 (**50**) could significantly reduce the expression of SGK3 and did not affect SGK1, SGK2, and S6K1. Other PROTACs had no good degradation effect and high selectivity for highly homologous SGK subtypes. Although there was no further study on the effect of E3 ligands on the selectivity of SGK subtypes, it can be concluded that when the POI ligands and linkers of PROTACs are uniform, the E3 ligands may play a decisive role in the high selectivity of the molecules.

**Figure 21:**
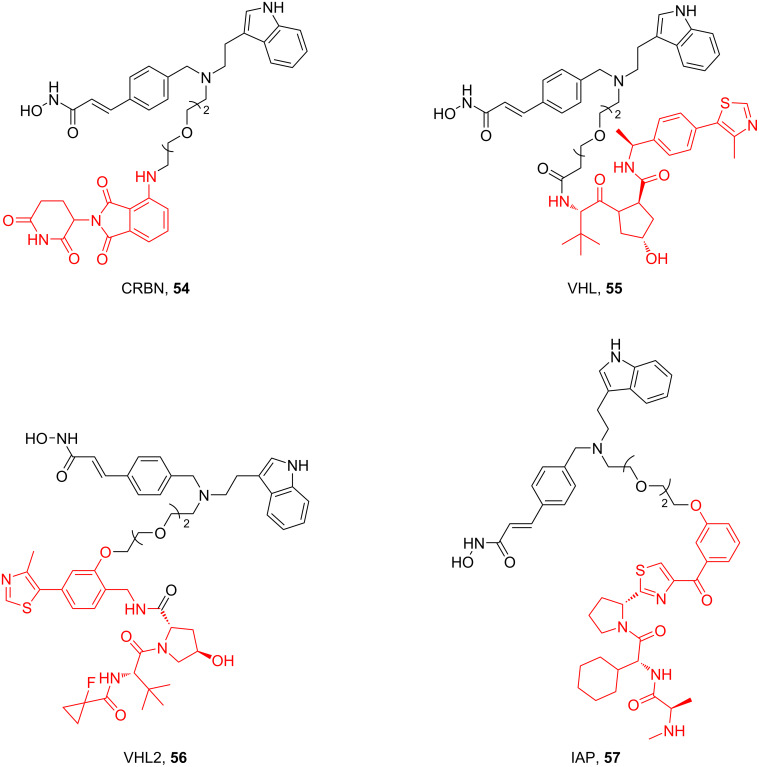
CRBN, VHL, and IAP ligands used when designing HDAC-PROTACs.

**HDAC:** Regarding the influence of linkers on the selectivity of PROTACs in highly homologous protein families, HDAC has been also a widely studied highly homologous protein family. Up to now, about 100 HDAC-PROTACs targeting CRBN, VHL, or IAP E3 ubiquitin ligases have been reported [87,137]. In 2021, Fischer et al. reported their studies on the difference between PROTACs based on CRBN, VHL, and IAP ligands for HDAC degradation (Figure 21). In addition, when designing PROTACs based on VHL ligands, they also studied the influence of two attachment points of VHL ligands on the molecular degradation effect [87]. It has been previously reported that the attachment point of the VHL ligand has a significant impact on the degradation characteristics of PROTACs [23]. In their study, by using the same HDAC inhibitors and linkers to focus only on the role of the recruited E3 ubiquitin ligase, they clarified that there are differences in degradation efficiency and substrate selectivity between PROTACs. They designed a group of PROTACs based on dacinostat. The results showed that PROTACs recruiting CRBN prefer to degrade HDAC6 and HDAC8 (**54**), while PROTACs based on VHL ligands strongly prefer to degrade HDAC3 (**55** and **56**). Differences in degradation efficiency were observed between two VHL-based degraders with only different attachment points of VHL the ligands, but degradation selectivity showed only slight differences. Interestingly, degraders based on IAP ligands showed weak but selective HDAC6 degradation (**57**).

The above is a brief list of recent studies on the vital influence of E3 ligands on the selectivity of PROTACs in highly homologous protein families. These studies showed that VHL ligands maybe have better selectivity than CRBN ligands in most cases. This view was put forward by Crews et al. as early as 2018 [138]. At that time, some studies showed that several PROTACs recruited CRBN could bind and degrade all BRD proteins bound by POI ligands [139–140]. However, when the PROTACs are designed with VHL ligands, it was proved that they can selectively degrade the target proteins [41,107]. To confirm this view, Crews et al. designed a series of PROTACs based on the c-Met tyrosine kinase inhibitor foretinib. Foretinib is a hybrid kinase inhibitor and proteomics has revealed that it can bind more than 130 kinases. When using foretinib as the POI ligand to connect with VHL ligand or CRBN ligand, respectively, through linkers, proteomic results show that the PROTACs recruiting CRBN and VHL have a significantly improved specificity compared with the individual POI ligand. The results showed that PROTACs recruiting CRBN could degrade 14 kinases, while PROTACs recruiting VHL could degrade 9 kinases. This shows that PROTAC technology can significantly improve the selectivity for target proteins compared with small-molecule inhibitors, and it seems that VHL ligands have better selectivity than CRBN ligands. Perhaps VHL ligands are an excellent choice for the development of PROTACs that specifically target highly homologous proteins in the future. However, this view still awaits further verification through additional research in the future.

#### E3 ligands with different structures target the same E3 ubiquitin ligases

As established by the above discussed studies utilizing different E3 ligands targeting different E3 ubiquitin ligases in PROTACs design can facilitate degradation selectivity. However, when considering the impact of E3 ligands on the selectivity of PROTACs, except considering the different types of E3 enzymes recruited by E3 ligands, it is also necessary to pay additional attention to the impact of changes in attachment sites on the selectivity of PORTACs when targeting the same E3 ubiquitin ligase.

**Figure 22:**
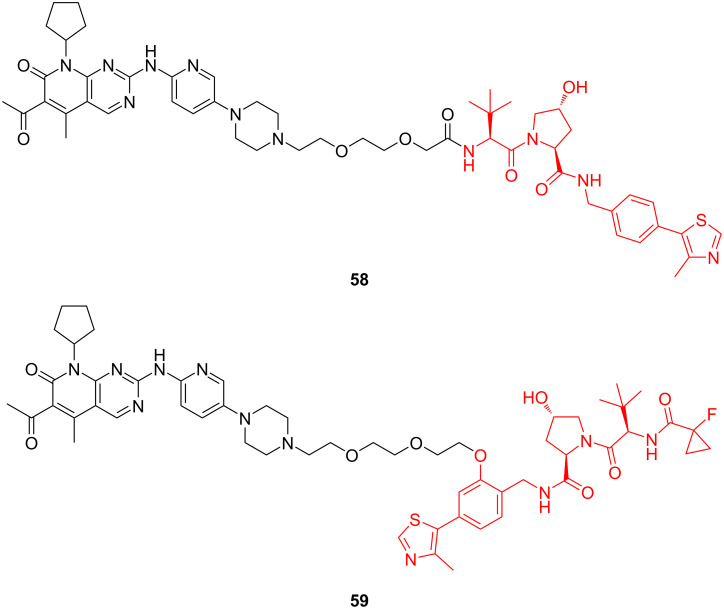
CDK4/6-PROTACs with different E3 ligands targeting the same E3 ubiquitin ligase.

In 2020, Gürschow, Krönke and co-workers conducted a study to explore the effect of E3 ligands on the selective degradation of CDK4 and CDK6 by PROTACs [131]. In this study, in addition to the study of different E3 ligands targeting CRBN and VHL, respectively, they also modified the structure of the VHL ligand and replaced its junction site with a linker, and finally synthesized compounds **58** and **59** (Figure 22). The results showed that compound **59** (DC_50 for CDK4_/DC_50 for CDK6 (at 0.1 μM)_ = 31) further enhanced the degradation activity compared with compound **58** (DC_50 for CDK4_/DC_50 for CDK6 (at 0.1 μM)_ = 7.4) and improved the ability to selectively degrade CDK6 compared with CDK4.

When talking about the HDAC protein family, we have introduced that Tang and co-workers investigated the design of PROTACs for selectively degrading HDAC based on CRBN ligands in 2019 [22]. They used nexturastat A as the selective inhibitor of HDAC6 with the CRBN E3 ligand pomalidomide to design 18 PROTACs. According to the different amino sites on the phthalimide ring of pomalidomide, these PROTACs were divided into two series. As for these compounds, the difference is the number of carbon atoms (*n*) between nexturastat A and the triazole ring or the number of carbon atoms (*m*) between pomalidomide and the triazole ring.

The results showed that the degradation effect of the C4-linker series PROTACs (Figure 7) was slightly more potent than that of the C5-linker series molecules (Figure 7), but the C5-linker series compounds demonstrated better selectivity than the C4-linker series compounds. This may be related to the different ubiquitination sites of HDAC6 by E3 ubiquitin ligase. Thus, as can be seen, in addition to the influence of the spatial distance caused by different linkers on the degradation efficiency and selectivity of PROTACs, a slight change in the connection position of the E3 ligands and linkers may also significantly affect the two properties.

In addition, as mentioned above, Crews et al. conducted a study to develop PROTACs that specifically degrade p38α and p38δ of the p38MAPK protein family [23]. In this study, they used foretinib as the POI ligand and two E3 ligands with different structures to target VHL together to explore the influence of the direction of VHL recruitment of PROTACs on selectivity (termed the “amide series” and “phenyl series” individually). Simultaneously, they used four linkers of different lengths to connect the E3 ligand and the POI ligand to explore the influence of linker fragments on molecular selectivity.

According to the results, it was found that in the two series of PROTACs, when the linker and the POI ligand are consistent, the molecular selectivity will also be widely different. For example, compound **26**, as example for the “amide series” PROTAC, shows no selectivity for p38MAPK protein family subtypes. In contrast, SJF-δ (**33**), as member of the “phenyl series” PROTAC, has a solid ability to degrade p38δ selectively (Figure 12).

At present, more studies still focus on the effect of linkers or different E3 ligands targeting different E3 ubiquitin ligases to design PROTACs with high degradation selectivity. There is still very little research on the selectivity of E3 ligands with different structures targeting the same E3 ubiquitin ligases to design different PROTACs. However, based on the above examples, it could be meaningful to study the effects of different structural types and connection sites targeting the same E3 ubiquitin ligases on the selectivity of PROTACs. When PROTACs are designed with the E3 ligands targeting the same E3 ubiquitin ligase, the replacement of the junction sites of E3 ligands and linker and the modification of E3 ligands may have unexpected effects on the selectivity of PROTACs. In the future, more research is needed in this field to better promote the development of PROTACs with good selectivity for members of highly homologous protein families.

### Influence of POI ligands on the selectivity of PROTACs in highly homologous protein families

As it is well-known, PROTACs are composed of three parts: POI ligand, linker, and E3 ligand. When designing PROTACs, research mainly focusses on the kind of linkers and E3 ligands and often ignores the POI ligands. Many studies use already approved small-molecule inhibitors or compounds under clinical research as POI ligands. This will lead to the effect of the POI ligands on the selectivity of PROTACs in highly homologous protein families, which is mainly determined by the selectivity of the small-molecule inhibitors used by POI ligands (Figure 17). However, multiple literature reports suggest that pairing the E3 ligase with the target protein is one of the most critical factors in generating potent and selective PROTACs [87,131,138,141]. This is primarily driven by the differential ability of varying E3 ligases to form a favorable ternary complex with a target POI [24]. Therefore, according to this view and after comparison and summary of a large number of studies, we are more convinced that the POI ligands are crucial to the selectivity of PROTACs.

In 2016, Crews et al. reported that the optimal pairing between the POI ligand and the recruited E3 ubiquitin ligase to achieve potent and selective PROTAC-induced target POI degradation is critical [126]. The important influence of E3 ligands on the selectivity of PROTACs highlighted in this study was already introduced above. This time, we look at this study from the perspective of POI ligands. In this study, the authors designed different PROTACs by connecting BCR-ABL TKI, which targets the binding of the c-ABL kinase domain, with a VHL ligand or CRBN ligand through the linker. Interestingly, according to the results, it is found that when dasatinib and bosutinib combine pomalidomide to recruit CRBN, the formed PROTACs can degrade both c-ABL and BCR-ABL. However, when they combined the VHL ligand to form PROTACs, the dasatinib-VHL PROTAC only degrades c-ABL and has no degradation effect on BCR-ABL. At the same time, the bosutinib-VHL PROTAC does not display any degradation activity.

Based on this study, it can be noticed that POI ligands can genuinely affect the selectivity of PROTACs. Although there are not many literature reports on the impact of POI ligands on the selectivity of PROTACs, many research groups have started to try to improve the selectivity of PROTACs through the optimization of POI ligands. In addition, it is worth noting that the effect of POI ligands on the selectivity of PROTACs is closely related to E3 ligands, which was well confirmed in this study. Moreover, it is believed that the impact of POI ligands is also inseparable from linkers. POI ligands, linkers, and E3 ligands, as the three parts of PROTACs, will influence and restrict each other to jointly regulate the selectivity of PROTACs.

### Influence of protein–protein interaction on the selectivity of PROTACs in highly homologous protein families

The mechanism of PROTAC-mediated target protein degradation intrinsically relies on the formation of a ternary complex [142]. This is a transient three-component assembly composed of the target protein, the PROTAC molecule, and the recruited E3 ubiquitin ligase. This process goes beyond the simple additive effects of the binary affinities between the POI ligand and its target, and between the E3 ligand and the ligase. A critical and often decisive factor governing the efficiency, stability, and selectivity of the ternary complex is the protein–protein interaction that arises between the POI and the E3 ligase under PROTAC-induced proximity [143]. The induced PPI interface can confer strong positive cooperativity, meaning that the affinity of ternary complex formation greatly exceeds that predicted from the individual binding events. In highly homologous protein families, whose members share highly conserved active or binding sites that limit the discriminative capacity of conventional small-molecule inhibitors, the geometric and chemical complementarity of the induced PPI interface becomes a powerful discriminator. Subtle conformational or surface electrostatic differences among homologous proteins can be amplified in the context of the ternary complex, enabling PROTACs to selectively engage and degrade one member rather than others. This PPI-driven selectivity represents a paradigm shift from traditional occupancy-based inhibition and offers a unique solution to long-standing challenges in drug discovery [144].

#### Molecular matching and spatial orientation

The ability of PROTACs to distinguish proteins with high sequence and structural similarity depends on precise molecular matching within the ternary complex [145]. Homologous proteins, such as kinases from the same family or bromodomains within the BET family, typically share conserved core folds and ligand-binding pockets. However, surface regions outside these canonical pockets that participate in protein–protein interactions may exhibit greater variability [146]. Through its specific linker length, rigidity, and attachment points, a PROTAC positions the POI and the E3 ligase in a unique spatial orientation. This orientation favors the formation of a specific PPI interface that may be optimal only for one member within a homologous family. For example, the surface of one subtype may present a cluster of complementary charges or hydrophobic residues that perfectly matches a region on an E3 ligase (such as VHL or CRBN), thereby forming a stable ternary complex [147–148]. A closely related subtype, although binding the POI ligand with similar affinity, may display a slightly different surface topology or charge distribution at this interface, resulting in weaker or non-productive PPIs, reduced ternary-complex stability, and consequently inefficient ubiquitination and degradation.

#### CDK

**Figure 23:**
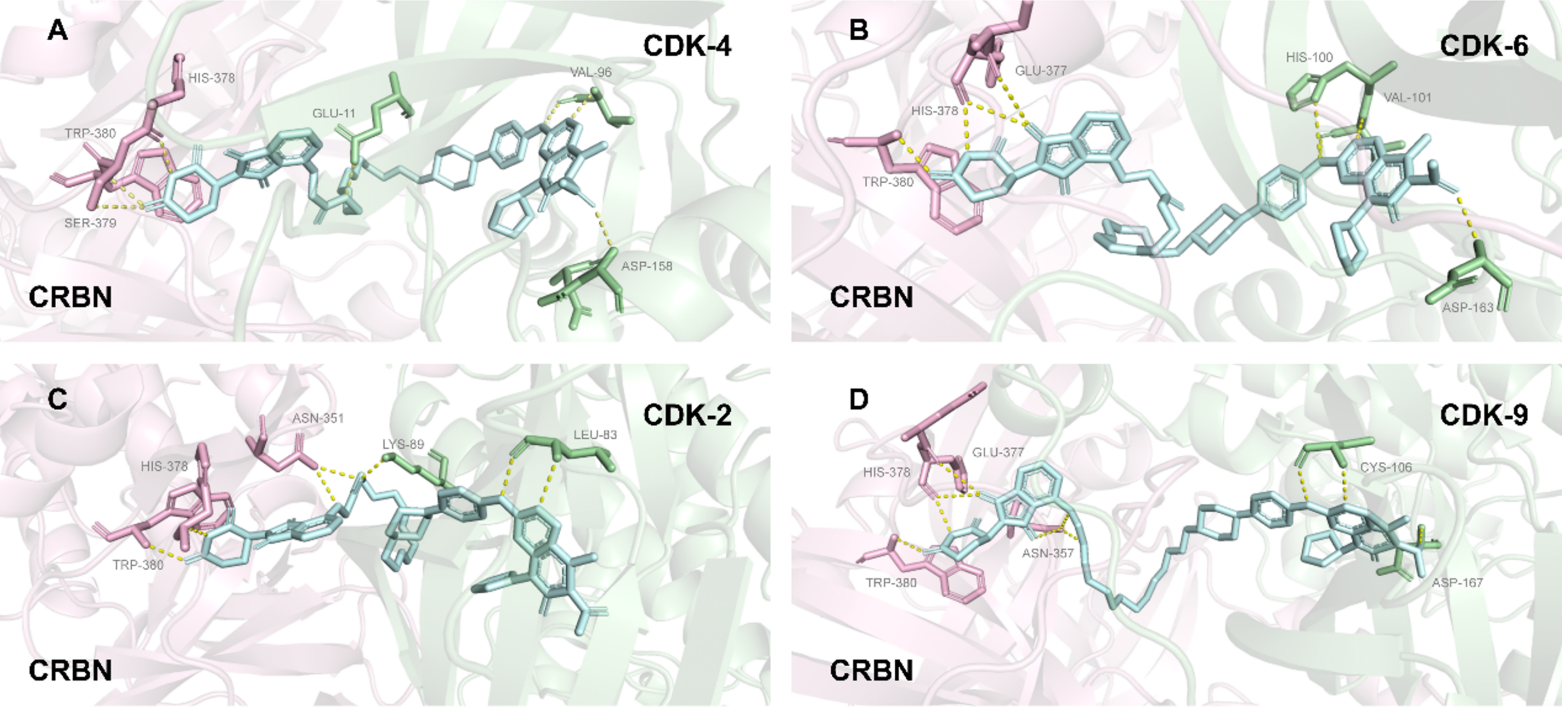
Structural basis for the selective degradation of CDK6 over CDK4 by PROTAC **2**. (A) CDK4–molecule **2**–CRBN complex. (B) CDK6–molecule **2**–CRBN complex. (C) CDK2–molecule **2**–CRBN complex. (D) CDK9–molecule **2**–CRBN complex.

For the CDK family, PROTAC molecules leverage the combinatorial design of their linkers and E3 ligands to shape finely differentiated PPI interfaces between homologous isoforms, thereby achieving a level of selectivity unattainable by traditional small-molecule inhibitors [149]. Taking CDK4 and CDK6 as examples, despite their 71% sequence homology, the PROTAC molecule **2** selectively degrades CDK6 while having no effect on CDK4 [34]. The fundamental reason is that this molecule, via a specific PEG linker, recruits CDK6 and the CRBN E3 ligase into a unique spatial conformation, forming a stable and catalytically active PPI interface. Although CDK4 can also bind to the same POI ligand (palbociclib), its surface cannot form a similarly compatible PPI interface with CRBN under the geometric configuration induced by molecule **2**, thus precluding effective ubiquitination (Figure 23).

Similarly, in the differentiation of CDK9 from CDK2, FN-1501-based PROTACs shift the degradation profile from dual CDK2/9 targeting to CDK9-specific selectivity by extending the linker length from 8–10 atoms to 11–12 atoms. This transition also stems from changes in the spatial orientation of the ternary complex caused by the variation in linker length: only the surface characteristics of CDK9 can form a complementary PPI interface with the E3 ligase under the new distance requirements, while CDK2 is excluded due to interface mismatch. These cases fully demonstrate that within homologous protein families, the spatial geometry regulated by the linker in a PROTAC determines the isoform-specific PPI interface, which in turn dictates selective degradation.

#### p38

**Figure 24:**
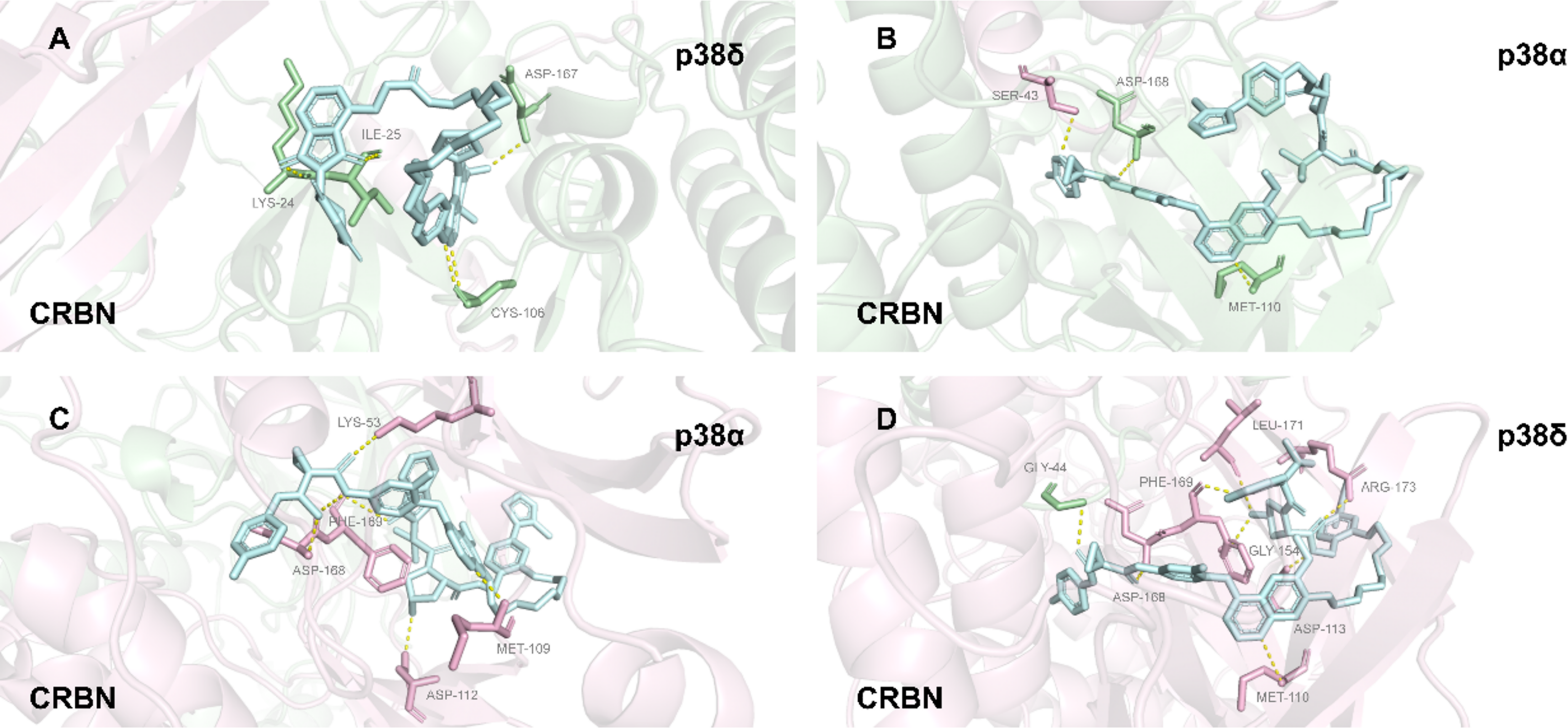
Regarding the PPI-driven selectivity mechanism of two PROTAC molecules, SJF-α and SJF-δ, for p38α and p38δ MAP kinases. (A) p38δ–molecule **29**–CRBN complex. (B) p38α–molecule **29**–CRBN complex. (C) p38α–molecule **33**–CRBN complex. (D) p38δ–molecule **33**–CRBN complex.

Within the highly homologous p38 MAPK family, the selective degradation capacity of PROTACs primarily depends on the stability and geometric configuration of PPIs within the induced ternary complexes. In 2019, Crews et al. utilized the promiscuous kinase inhibitor foretinib as a warhead [23], combined with two VHL ligands featuring distinct linkage modes (amide vs phenyl series) and varying linker lengths, to develop two PROTAC molecules with orthogonal selectivity: molecule **29** (13-atom linker, amide linkage) and molecule **33** (10-atom linker, phenyl linkage). Although both molecules can bind to both p38α and p38δ, molecule **29** selectively degrades p38α (DC_50_ = 7.16 nM, D_max_ = 97.4%) while exhibiting almost no degradation of p38δ (D_max_ = 18%). Conversely, molecule **33** selectively degrades p38δ (DC_50_ = 46.17 nM, D_max_ = 99.4%) and has no effect on p38α. To investigate the mechanism underlying this differential selectivity, the authors performed in vitro GST-pulldown assays, which revealed that molecule **29** efficiently enriched p38α to form a ternary complex while molecule **33** did not. However, for p38δ, both PROTACs were able to induce ternary complex formation, suggesting that the mere presence of a ternary complex is not a sufficient condition for degradation. Further determination of the affinity and kinetics of the p38δ–PROTAC–VHL ternary complexes using surface plasmon resonance (SPR) showed that the complex induced by SJF-δ (**33**) possessed stronger affinity (*K*_d_ = 436 nM) and a longer dissociation half-life (*t*_1/2_ = 38 s). In contrast, the complex induced by SJF-α exhibited weaker affinity (*K*_d_ = 1.2 μM) and a shorter half-life (*t*_1/2_ = 8 s). Ternary complex capture assays in cell lysates also confirmed that SJF-δ could stably enrich endogenous p38δ, whereas SJF-α could not. Molecular dynamics simulations indicated that the two PROTACs guide VHL to dock with p38δ in different conformations: in the presence of molecule **33**, Arg108 of VHL forms favorable electrostatic interactions with Glu49/Glu160 of p38δ; however, in the presence of molecule **29**, Arg108 makes unfavorable contacts with Lys220/Thr221 of p38δ. To validate this prediction, the authors constructed a K220E/T221E double point mutant of p38δ. When expressed in cells, the mutant p38δ, which was originally resistant to molecule **29**, became effectively degradable by molecule **29**. This result directly demonstrates that subtle differences in the PPI interface within the ternary complex dictate the selectivity of PROTACs for highly homologous proteins, providing a theoretical foundation for the future design of precision degraders based on PPI optimization (Figure 24).

#### STAT

The members of the STAT (signal transducer and activator of transcription) family exhibit high structural homology, particularly within the SH2 domain [150].

**Figure 25:**
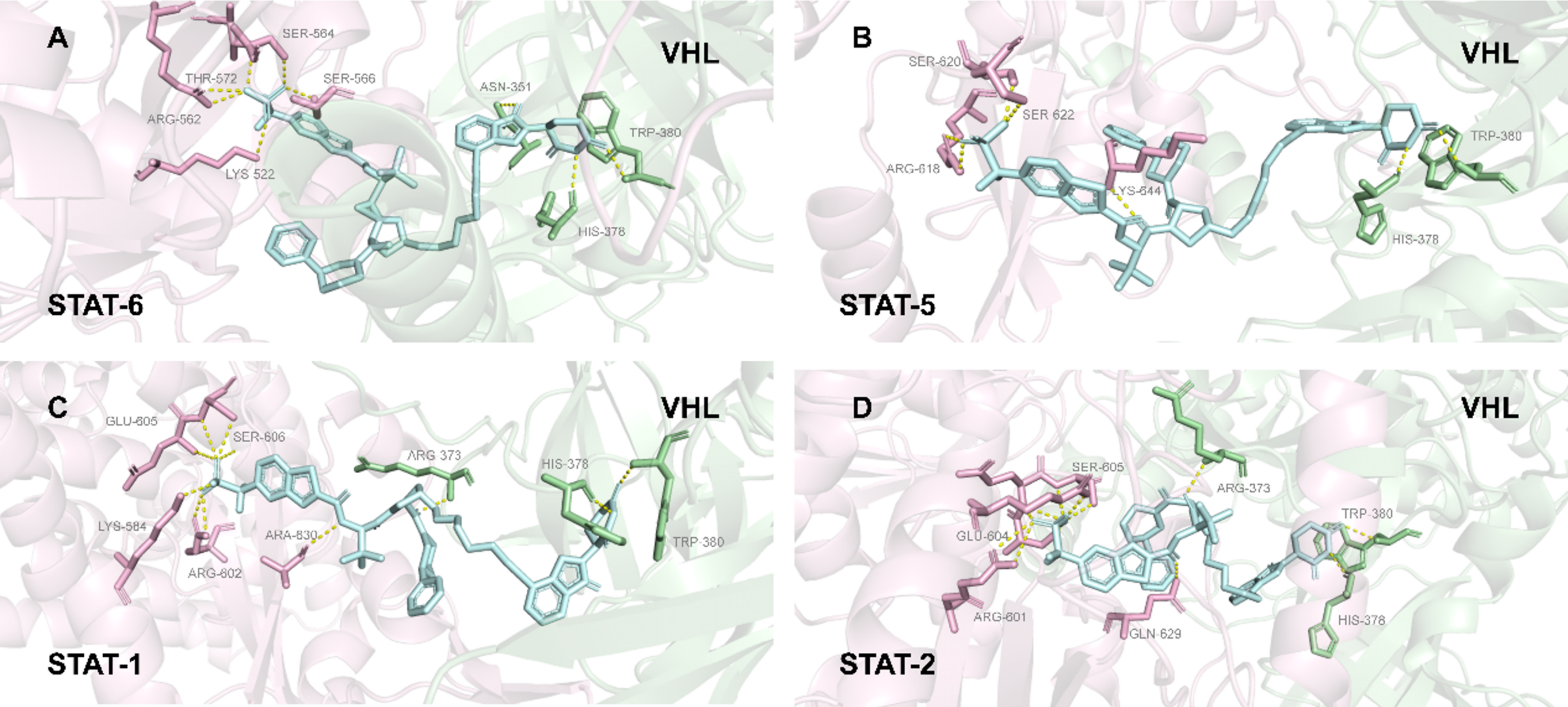
Co-crystal structure and ultra-high selectivity of the STAT6 PROTAC degrader AK-1690. (A) STAT6–AK-1609–CRBN complex. (B) STAT5–AK-1609–CRBN complex. (C) STAT1–AK-1609–CRBN complex. (D) STAT2–AK-1609–CRBN complex.

In 2024, Wang and co-workers reported the discovery of AK-1690, the first potent and highly selective STAT6 PROTAC degrader [151]. The breakthrough of this study lies first in the optimization and acquisition of the ligand AK-068, which possesses an extremely high affinity for STAT6 (*K*_i_ = 6 nM) and >85-fold selectivity over STAT5. Based on this ligand, the researchers designed the PROTAC molecule AK-1690. Experimental data demonstrated that AK-1690 effectively induces STAT6 protein degradation in cells (with a DC_50_ as low as 1 nM), while showing almost no degradation effect on other STAT family members, including STAT1, STAT2, STAT3, and STAT5, at concentrations as high as 10 μM. The researchers successfully resolved the first co-crystal structure of STAT6 in complex with AK-1690, providing a structural basis for understanding its ultra-high selectivity. This structure revealed a precise complementary relationship at the binding interface between AK-1690 and STAT6. Although the ligand binding pocket of STAT6 shares some similarities with other STAT members, the ternary complex interface formed by AK-1690 and STAT6 contains numerous unique, non-conservative interactions (Figure 25). These PROTAC-induced interprotein contacts render the entire complex highly sensitive to the surface topology and amino acid residue characteristics of STAT6, thereby excluding other family members and achieving exceptional selectivity.

The protein–protein interactions within PROTAC-induced ternary complexes constitute a foundational mechanism for achieving unprecedented selectivity against highly homologous protein families. It goes beyond simple target engagement and instead exploits subtle biophysical complementarity between two proteins [145]. This selectivity arises from the cooperative interplay among the POI ligand, the linker, and the E3-ligase ligand. Quantitative frameworks for cooperativity, the explanatory power of structural biology, and the predictive capacity of computational modeling are transforming PROTAC design from empirical screening into a more rational, structure-guided endeavor. Even when using the same or similar POI ligands, the degradation profile of PROTACs against homologous proteins can be significantly altered by optimizing the choice of E3 ligase ligand, the length of the linker, and the attachment site (Table 2). Future advances will depend on discovering new E3-ligase ligands to expand the “PPI toolbox,” obtaining more ternary-complex structures across diverse target families, and improving computational algorithms to accurately predict cooperative binding [152]. By harnessing the principles of PPI-driven selectivity, the PROTAC paradigm holds promise for delivering highly precise therapeutics capable of discriminating between “sibling” proteins, thereby minimizing off-target effects and opening new therapeutic avenues for diseases driven by specific members of redundant protein families [153].

**Table 2:** Summary of representative PROTAC molecules and their degradation activities.

Molecule name (compound number)	E3 ligase	Target (POI)	Data values

PROTAC-1 (**1**)	CRBN	CDK4/6	in Jurkat cells: CDK4 degradation rate >50% (0.1 μM); CDK6 degradation rate >95% (0.1 μM)
BSJ-03-123 (**2**)	CRBN	CDK6	it exhibits superior degradation capability towards CDK6, while proteomic results indicate no degradation effect on CDK4
PROTAC-6 (**4**)	CRBN	CDK6	selectively degrading CDK6 in HPNE and MiaPaCa2 cells does not affect CDK2, 4, 5, 7, 9
PROTAC-pal-pom (**5**)	CRBN	CDK4/6	MDA-MB-231cells: DC_50_(CDK4) = 13 nmol/L; DC_50_(CDK6) = 34 nmol/L
PROTAC-CP 10 (**6**)	CRBN	CDK6	U251 cells: DC_50_(CDK6) = 2.1 nmol/L; DC_50_(CDK4) > 100 nmol/L
compounds **9**–**11**	CRBN	CDK2/9	it is a dual degrader for CDK2/9
compounds **12**–**14**	CRBN	CDK9	selective degradation of CDK9
compound **16c**	CRBN	CDK9	it exhibits optimal CDK9 degradation activity and high selectivity towards CDK9 compared to CDK5
compound **19c**	CRBN	HDAC6	it exhibits the best selective degradation effect on HDAC6 compared to HDAC1, 2, and 4
compound **20d**	CRBN	HDAC6	DC_50_ = 1.64 ± 0.24 nM
compound **20h**	CRBN	HDAC6	at a concentration of 100 nM, it can degrade 78.3–80.1% of HDAC6
compound **20i**	CRBN	HDAC6	at a concentration of 100 nM, it can degrade 82.1–84.1% of HDAC6
compound **21j**	VHL	HDAC6	DC_50_ = 7.1 nM (exhibiting the best selectivity compared to HDAC1, 2, 3, 4, 7, 8)
MZ1 (**35**)	VHL	BRD4	it exhibits excellent selective degradation capability for BRD4 (compared to BRD2 and BRD3)
SJF-8240/SJF-α (**29**)	VHL	p38α	DC_50_(p38α) = 7.16 ± 1.03 nM; D_max_ = 97.4%
compound **33**	VHL	p38δ	DC_50_(p38δ) = 46.17 ± 9.85 nM; D_max_ = 99.41 ± 3.31%
SHD913 (**37**)	VHL	BRD4	in PC-3 cells: BRD4 long isoform DC_50_ = 7.7 nM, short isoform DC_50_ = 5.0 nM

## Conclusion

To sum up, this review mainly introduces the advantages of PROTAC over small-molecule inhibitors in the selectivity of highly homologous protein families. According to the systematic summary, we can clearly find that both linker and E3 ligand are crucial to the selectivity of PROTAC design (Table 3). Whether it is an E3 ligand or a linker fragment, it may ultimately affect the overall structure of the PORTAC to achieve highly selective degradation through the subtle differences between PPI and ubiquitinated lysine sites. Meanwhile, the selective degradation of highly homologous protein families by PROTACs underscores the fundamental pharmacological shift from occupancy-driven inhibition to event-driven target elimination. This shift allows selectivity to emerge from ternary complex cooperativity, induced protein–protein interactions, and spatial orientation, rather than solely from binding affinity differences.

**Table 3:** Factors influencing the selectivity of PROTACs in highly homologous protein families.

protein level	surface lysine availability	the presence and spatial accessibility of specific lysine residues on the protein surface that are required for ubiquitination
	surface topology & residues	unique biophysical features and amino acid residues (even in highly homologous proteins) that facilitate or hinder specific protein-protein interactions (PPIs)
	tissue and cell expression	differential abundance of the target protein or the recruited E3 ligase across different tissues and cell types
	subcellular localization	the specific cellular compartment where a protein resides can determine its accessibility to the PROTAC and the degradation machinery
	protein–protein complexes	target proteins existing within multiprotein complexes can have different "degradability" compared to their monomeric forms

PROTAC level	POI warhead selection	the choice of the ligand for the target protein (POI) and its specific binding orientation or "exit vector"
	E3 ligase recruitment	selecting different E3 ligases (e.g., VHL vs CRBN) can drastically alter the degradation profile of the same target
	linker engineering	optimization of the linker’s length, flexibility, and chemical composition (e.g., PEG vs alkyl chains) to allow for productive orientation
	linker attachment points	the specific positions on the ligands where the linker is attached, which determines the spatial orientation of the recruited proteins
	ternary complex stability	the ability of the PROTAC to induce stable and **cooperative** de novo protein–protein interactions between the POI and the E3 ligase

The difference between protein–protein interaction and ubiquitinated lysine sites is currently widely accepted as two factors affecting the selectivity of PROTAC molecules in highly homologous protein families. It is generally accepted that the degradation effect of PROTACs requires the formation of ternary complexes with POI and E3 ubiquitin ligase. However, before Cullin et al. published their research in 2017, few people believed that when PROTAC formed ternary complexes, the protein–protein interaction between E3 ubiquitin ligase and POI PPI would have a huge impact on the degradation of PROTAC molecules, and even affect the selectivity of PROTAC molecules to highly homologous protein families. The existence of the PPI effect makes the formation of ternary complexes not only depend on the affinity between POI ligand and POI but also between E3 ligand and E3 ubiquitin ligase, which further confirms that there is no inevitable relationship between the binding affinity of PROTAC molecules and the degradation effect. The existence of the PPI effect makes it easier or more difficult to form ternary complexes among members of the highly homologous protein family with only slight structural differences, thus enabling PROTAC molecules to achieve selectivity between different subtypes. This is a milestone for the future development of highly specific PROTAC molecules [154]. On this basis, in order to explore more about the formation of ternary complexes, it is essential to analyze the crystal structure of ternary complexes as much as possible to explore the mechanism by which a PROTAC plays its high selectivity in highly homologous protein families. Therefore, in recent years, many researchers have devoted themselves to building corresponding prediction models using computer simulations to accurately predict the crystal structure of PROTAC ternary composites, so as to better develop PROTAC molecules for various targets in the future [155]. Furthermore, following ternary complex formation, PROTACs promote the ubiquitination of the POI by recruiting the E3 ubiquitin ligase, thereby triggering its proteasomal degradation. Despite the high sequence similarity within certain protein families, differences in the accessibility and positioning of lysine residues result in distinct ubiquitination patterns. This provides an additional layer of selectivity for PROTAC-mediated degradation. Although this concept has been proposed, systematic investigations validating the relationship between lysine site distribution and degradation selectivity remain limited. Future studies in this area are therefore warranted [154].

Looking forward, stimulus-responsive degraders such as light-controllable PROTACs (opto-PROTACs) may further expand the capabilities of event-driven pharmacology by integrating structural precision with spatial and temporal regulation. Conditional activation strategies could ultimately enhance selectivity among highly homologous proteins by restricting degradation to defined biological contexts.

## Data Availability

Data sharing is not applicable as no new data was generated or analyzed in this study.
